# Artificially engineered antiferromagnetic nanoprobes for ultra-sensitive histopathological level magnetic resonance imaging

**DOI:** 10.1038/s41467-021-24055-2

**Published:** 2021-06-22

**Authors:** Zeyu Liang, Qiyue Wang, Hongwei Liao, Meng Zhao, Jiyoung Lee, Chuang Yang, Fangyuan Li, Daishun Ling

**Affiliations:** 1 Institute of Pharmaceutics and College of Pharmaceutical Sciences, Zhejiang University, 310058 Hangzhou, China; 2 Hangzhou Institute of Innovative Medicine, College of Pharmaceutical Sciences, Zhejiang University, 310058 Hangzhou, China; 3 School of Chemistry and Chemical Engineering, Frontiers Science Center for Transformative Molecules, National Center for Translational Medicine, Shanghai Jiao Tong University, 200240 Shanghai, China

**Keywords:** Cancer imaging, Imaging techniques and agents

## Abstract

Histopathological level imaging in a non-invasive manner is important for clinical diagnosis, which has been a tremendous challenge for current imaging modalities. Recent development of ultra-high-field (UHF) magnetic resonance imaging (MRI) represents a large step toward this goal. Nevertheless, there is a lack of proper contrast agents that can provide superior imaging sensitivity at UHF for disease detection, because conventional contrast agents generally induce T2 decaying effects that are too strong and thus limit the imaging performance. Herein, by rationally engineering the size, spin alignment, and magnetic moment of the nanoparticles, we develop an UHF MRI-tailored ultra-sensitive antiferromagnetic nanoparticle probe (AFNP), which possesses exceptionally small magnetisation to minimize T2 decaying effect. Under the applied magnetic field of 9 T with mice dedicated hardware, the nanoprobe exhibits the ultralow *r*_*2*_/*r*_*1*_ value (~1.93), enabling the sensitive detection of microscopic primary tumours (<0.60 mm) and micrometastases (down to 0.20 mm) in mice. The sensitivity and accuracy of AFNP-enhanced UHF MRI are comparable to those of the histopathological examination, enabling the development of non-invasive visualization of previously undetectable biological entities critical to medical diagnosis and therapy.

## Abstract (web summary)

Ultra-high-field (UHF) magnetic resonance imaging (MRI) has potential for imaging disease including cancer metastasis. Here, the authors develop an ultra-sensitive antiferromagnetic nanoparticle probe with a small magnetisation for use in UHF MRI and demonstrate the ability to detect small primary tumours and micrometastases in mice.

## Introduction

Despite the recent development of molecular imaging, histopathological examination remains the gold standard for clinical tumour diagnosis, which can clearly distinguish tumours from the normal tissue with high contrast^1–4^. The histopathological examination involves invasive procedures including biopsy or surgical resection, which inevitably has many drawbacks such as being time-consuming and the risk of causing complications^5–7^. Therefore, the ultimate goal of non-invasive imaging approaches is to obtain histopathological level imaging performance. Current imaging modalities, such as magnetic resonance imaging (MRI), positron emission tomography (PET), single-photon emission computed tomography (SPECT) and computed tomography (CT), are being actively employed in non-invasive clinical diagnosis^8–10^. However, PET and SPECT cannot provide anatomical information and have poor spatial resolutions^10^, while CT faces the disadvantage of limited soft-tissue contrast^11,12^. MRI is a powerful diagnostic technique that can be used to acquire structural and anatomical details for disease diagnosis^8,13–15^. Currently, the majority of clinical MRI machines are installed with a low magnetic field (1.5 T or 3 T), which can only resolve anatomical structures of 1 mm in size^16^. In principle, high magnetic field strength and long scanning time both can improve the signal-to-noise ratio (SNR) and thus allow the acquisition of images with higher resolution^17^. Recently, the development of ultra-high-field (UHF) MRI system (≥7 T) has raised the hope to visualise microscopic biological objects that are beyond the capacity of low-field MRI, such as the microvasculature as small as 100 μm in diameter^18^. Although UHF MRI provides high spatial resolution, the long scanning time and the longer time required for UHF MRI, hamper patient compliance in clinical practice^18^. Moreover, the susceptibility artefact induced by contrast agent^15^ and the prolonged T1 relaxation times of water protons at UHF^18^ would lead to the loss of T1 contrast between adjacent tissues.

The sensitivity and the image resolution of UHF MRI needs to be improved by using contrast agents, which selectively shorten the relaxation times in the region of interest (ROI) and thus enhance the SNR of ROI^19,20^. Current MRI contrast agents are often paramagnetic metal chelates^21–24^ or metal oxide nanoparticles^13–15,25–28^, which can accelerate the T1 relaxation (the recovery of the net magnetisation in the longitudinal direction (**M**_**z**_)) of water protons for T1-weighted MRI contrast enhancement^14^. Particularly, nanoparticle-based contrast agents with a large surface area can be easily modified with targeting ligands (e.g., antibodies and peptides), for targeted delivery and locally amplified MRI^29–33^. Upon application of an external field, the magnetic moments of these contrast agents tend to align in the direction of external field^34^, and their magnetisations increase substantially with the external magnetic field strength (*M* = *χB*_*0*_, where *M*, *χ* represent the magnetisation and magnetic susceptibility of contrast agent, and *B*_*0*_ represent external magnetic field strength, respectively)^15,26^, resulting in the enhancement of T2-decaying effects^35^. In principle, for T1-weighted imaging, the recovered **M**_**z**_ should be flipped to the transverse (*xy*) plane after the excitation with a 90° radiofrequency pulse (RF), which renders it susceptible to the contrast agent-induced T2-decaying effect and the resultant quick dephasing of **M**_**z**_, eventually attenuating the signal on T1-weighted images^13,35^. At UHF MRI, conventional paramagnetic and superparamagnetic probes would diminish the recovery of **M**_**z**_ and exhibit poor T1 contrast capability due to the strong T2-decaying effects. Consequently, the development of imaging probes that are tailored to the UHF platform is challenging but it is crucial to fully realising the sensitivity of UHF MRI.

**Fig. 1:**
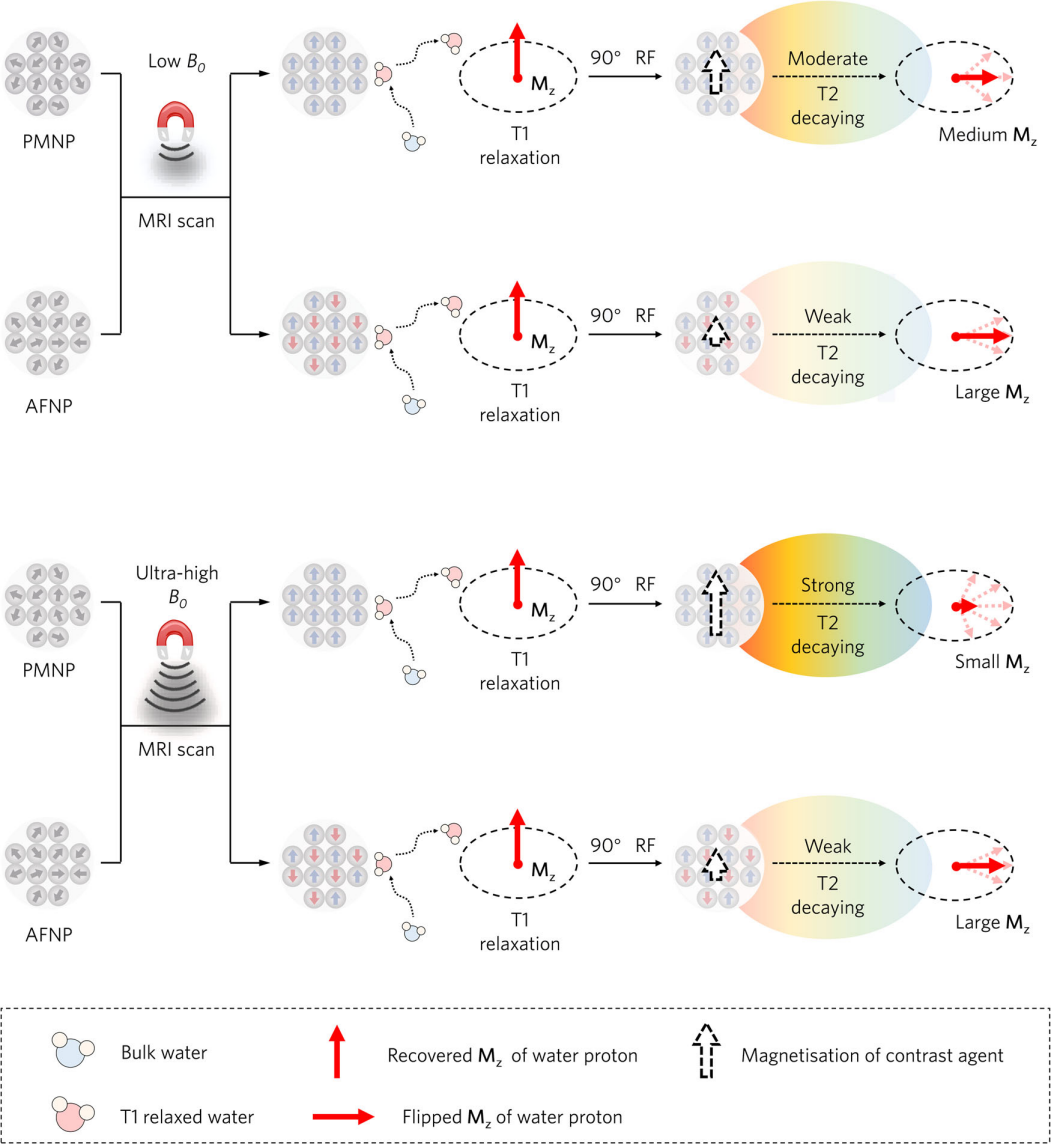
Design principle of nanoprobes for UHF MRI. The recovery of **M**_**z**_ using nanoprobe at MRI scans with different external *B*_*0*_. At room temperature and in the absence of a magnetic field, the magnetic moments of PMNPs or AFNPs are both randomly aligned. When a field is imposed, the magnetic moments of PMNPs tend to align unanimously in the direction of the field, leading to a high magnetisation, whereas the magnetic moments of AFNPs are aligned in antiparallel directions and equal in magnitude, resulting in a small magnetisation. Therefore, AFNPs have a weaker T2-decaying effect compared with PMNPs at UHF MRI, and thus promote the recovery of **M**_**z**_, leading to a larger **M**_**z**_ and subsequent better T1 contrast efficiency.

Based on the aforementioned principles, artificially decreasing the magnetisation of contrast agents may mitigate their strong T2-decaying effect in UHF MRI, which is crucial to **M**_**z**_ recovery required for T1-weighted imaging. Herein, we report antiferromagnetic nanoparticle probes (AFNPs) harbouring antiparallel magnetic moments, which are refractory to the external magnetising field as compared with paramagnetic nanoparticle probes (PMNPs) (Fig. 1). Using a 9-T UHF MRI scanner, we systemically investigate the UHF MRI performance of a series of AFNPs and PMNPs with different particle sizes, spin alignment modes and magnetic moments, and develop an optimised antiferromagnetic nanoprobe that affords satisfactory MRI contrasting ability at UHF. Once conjugated with the cyclic arginyl–glycyl–aspartic acid (RGD) peptide (cRGDyK) for neovasculature targeting, these antiferromagnetic nanoprobes enable UHF MRI to detect primary small tumours and micrometastases with high sensitivity and accuracy, providing detailed information about the microscopic tumours as small as 0.20 mm, which to date could only be revealed by histopathological examination. We anticipate the antiferromagnetic nanoprobes as demonstrated herein are ideal candidates for the future development of UHF-competent high-performance contrast agents, to maximise the utility of the next-generation UHF MRI for the visualisation of previously undetectable biological lesions.

## Results

### Development of ultra-sensitive nanoprobes for UHF MRI

**Fig. 2:**
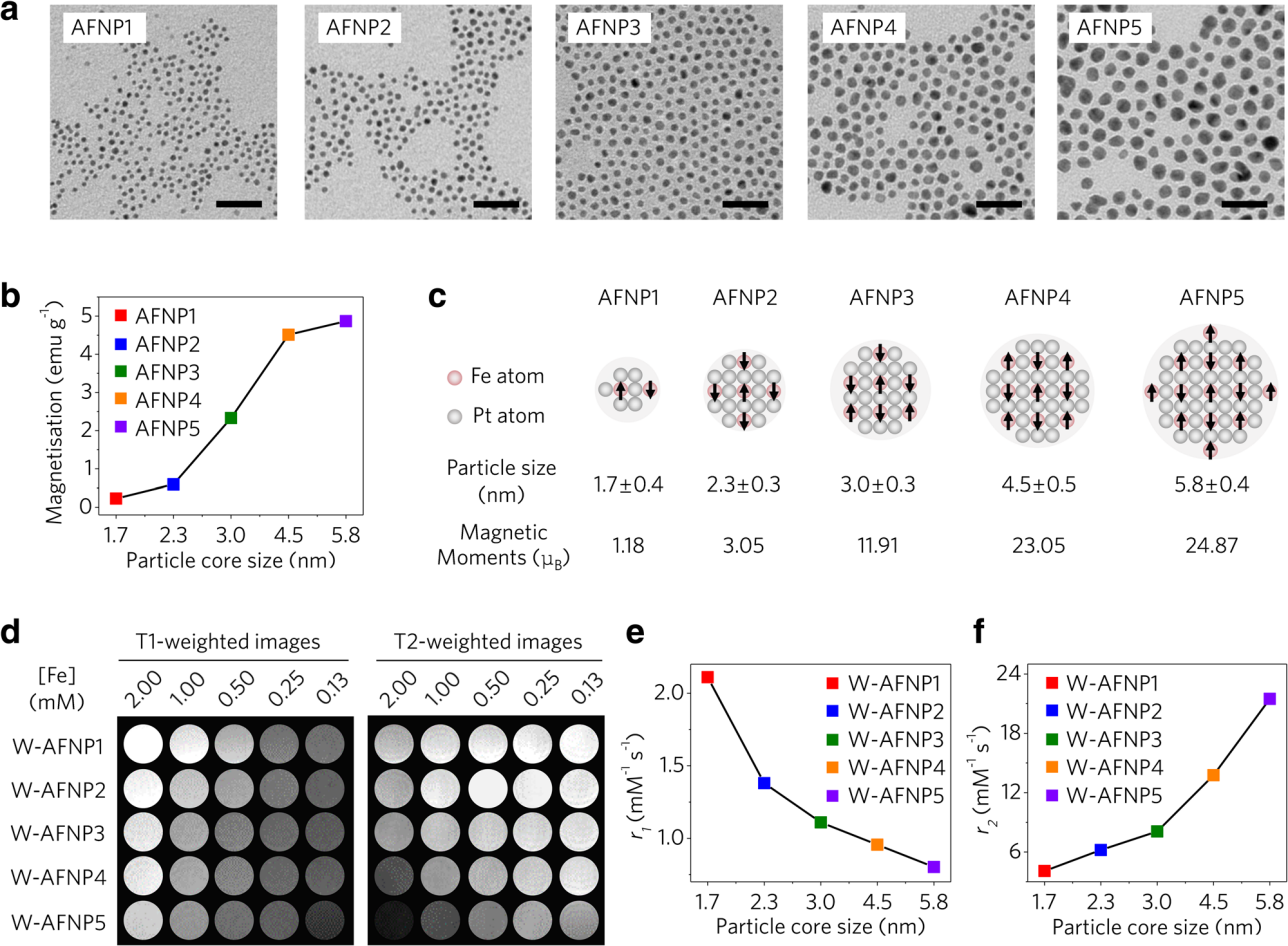
Size-dependent UHF MRI performance of nanoprobes. **a** Transmission electron microscopy (TEM) images of AFNP1, AFNP2, AFNP3, AFNP4 and AFNP5, scale bar = 20 nm. **b** Magnetisation values of AFNP1, AFNP2, AFNP3, AFNP4 and AFNP5 at 5 T measured at 300 K. **c** Schematics of magnetic moments for different-sized AFNPs. **d** T1-weighted and T2-weighted MR images of W-AFNP1, W-AFNP2, W-AFNP3, W-AFNP4 and W-AFNP5 in aqueous solution at various Fe concentrations (mM). **e**, **f** Comparison of (**e**) *r*_1_ values and (**f**) *r*_*2*_ values of W-AFNP1, W-AFNP2, W-AFNP3, W-AFNP4 and W-AFNP5. In **a**, experiments were repeated three times independently. Source data are provided as a Source Data file.

A series of uniform-sized AFNPs (AFNP1, AFNP2, AFNP3, AFNP4 and AFNP5) with different core diameters (1.7 ± 0.4, 2.3 ± 0.3, 3.0 ± 0.3, 4.5 ± 0.5 and 5.8 ± 0.4 nm, respectively) were synthesised by thermal decomposition of iron and platinum acetylacetonates in the presence of oleic acid and oleylamine as surfactants (Fig. 2a)^36^. X-ray diffraction (XRD) patterns reveal that different-sized AFNPs have identical FePt_3_ (JCPDS no. 29-0716) crystalline structures (Supplementary Fig. 1). The Fe/Pt molar ratios of AFNPs are determined to be ~1:3 by inductively coupled plasma-mass spectrometry (ICP-MS) (Supplementary Table 1). X-ray photoelectron spectroscopy (XPS) analysis shows there are ferric ions (Fe^3+^) on the surface of AFNPs (Supplementary Fig. 2). The magnetisation values of AFNPs at 5 T tend to decrease as the particle size decreases (Fig. 2b). According to the equation *μ*_*sp*_ = 4/3π*Mρr*^3^ (*μ*_*sp*_, *ρ* and *r* are the magnetic moments, density and core size of the nanoparticle, respectively)^37^, the magnetic moments at room temperature for AFNPs are calculated to be ~1.18, 3.04, 11.91, 23.05, 24.87 μ_B_, respectively, indicating that smaller-sized nanoparticles possess a lower magnetic moment (Fig. 2c). For the MRI study, AFNPs were transferred to an aqueous medium (Supplementary Fig. 3) via ligand exchange with carbonylated poly (ethylene glycol)^38^ and their longitudinal relaxivity (*r*_1_) and transverse relaxivity (*r*_2_) values were measured using a 9-T MRI scanner. The water-dispersible AFNP1 (W-AFNP1) exhibits the brightest signals among all groups in MRI phantom images (Fig. 2d), with the highest *r*_1_ value (2.11 mM^−1^ s^−1^), lowest *r*_2_ value (4.08 mM^−1^ s^−1^), and smallest *r*_2_/*r*_1_ value (~1.93) (Fig. 2e, f and Supplementary Fig. 4), and were used for further studies.

**Fig. 3:**
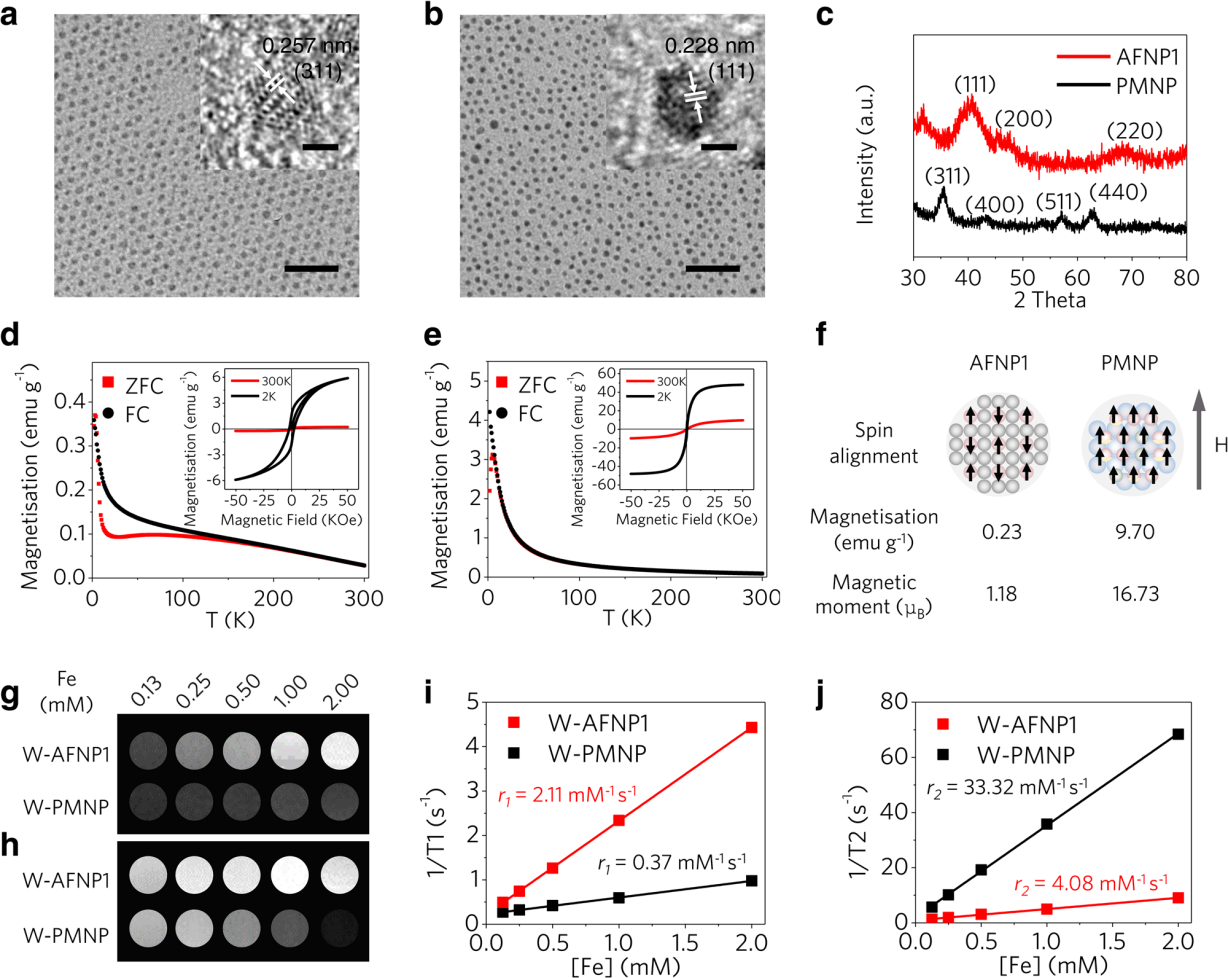
Effect of magnetic moments and spin alignments of nanoprobes on UHF MRI performance. **a**, **b** TEM images of (**a**) PMNP and (**b**) AFNP1, scale bar = 20 nm. The inset images in panels **a** and **b** are the high-resolution transmission electron microscopy (HRTEM) images of PMNP and AFNP1, respectively, scale bar = 1 nm. **c** XRD patterns of AFNP1 (FePt_3_; JCPDS no. 29-0716) and PMNP (γ-Fe_2_O_3_; JCPDS no. 39-1346). **d**, **e** Temperature-dependent magnetisation curves (*M*–*T*) for (**d**) AFNP1 and (**e**) PMNP, measured after ZFC and FC at the applied field of 100 Oe. Their inset images show the *M*–*H* curves at 2 K and 300 K for (**d**) AFNP1 and (**e**) PMNP. **f** Schematics of spin alignments of magnetic components at the external magnetic field, as well as the magnetic moments and magnetisation values of AFNPs and PMNPs (red circles, grey circles and blue circles represent the Fe atoms, Pt atoms and oxygen atoms, respectively). **g** T1-weighted and **h**, T2-weighted MR images of W-AFNP1 and W-PMNP. **i** T1 relaxation rate (1/T1, s^−1^) and **j** T2 relaxation rate (1/T2, s^−1^) plotted against Fe concentration (mM) for W-AFNP1 and W-PMNP. In **a**, **b**, experiments were repeated three times independently. Source data are provided as a Source Data file.

Small-sized iron oxide nanoparticles^14,28,37^ with a core diameter of 1.8 ± 0.5 nm were fabricated as a typical PMNP (Fig. 3a) for comparison with AFNP1 (Fig. 3b, c). The zero-field-cooled (ZFC) and field-cooled (FC) curves of AFNP1 show a bifurcation when the temperature drops below ~200 K, and the ZFC curve displays a peak-like feature at ~75 K (Fig. 3d). The field-dependent magnetisation (*M*–*H*) curve of AFNP1 measured at 300 K gives an ultralow magnetisation (0.23 emu g^−1^) at 5 T and shows no coercivity and remanence (Fig. 3d, inset). When measured at 2 K, a slight magnetic hysteresis is observed in the *M*–*H* curve of AFNP1 (Fig. 3d, inset), demonstrating the antiferromagnetic behaviour of AFNP1^39,40^. In contrast, the ZFC curve of PMNP deviates from the FC curve at a low temperature of ~6 K (Fig. 3e). Compared with AFNP1, PMNP exhibits a much higher magnetisation (9.70 emu g^−1^) at 5 T with negligible coercivity and remanence (Fig. 3e, inset). In theory, the sublattices of magnetic spins in the antiferromagnetic structure are antiparallel, resulting in an ultralow magnetisation of AFNP1^34,41^ (Fig. 3f). Notably, comparing with water-dispersible PMNP (W-PMNP), W-AFNP1 shows significantly brighter T1-weighted phantom images with an approximately sixfold higher *r*_1_ value (Fig. 3g–j and Supplementary Fig. 5).

We further investigated the mechanism for the UHF MRI contrast performance of AFNP1. In principle, the MRI signal intensity (*I*) obeys the following function^14^,

1 $$I={M}_{0}\,{{\exp }}\left(-\frac{{T}_{E}}{{T}_{2}}\right)\left[1-{{\exp }}\left(-\frac{{T}_{R}}{{T}_{1}}\right)\right]$$

where *T*_*R*_ and *T*_*E*_ are sequence parameters that represent repetition time and echo time, while *T*_2_ and *T*_1_ represent transverse and longitudinal relaxation times of water protons, respectively. As shown in Eq. (1), MRI contrast agents enhance the signal intensity in the ROI by significantly shortening *T*_1_ and slightly shortening *T*_2_. On the one hand, the particle surface Fe^3+^ with five unpaired electrons can effectively accelerate the T1 relaxation process of surrounding water protons^37^. On the other hand, for contrast agents in the motional averaging regime, their contribution to *T*_2_ can be given as follow^14,19,42,43^,

2 $$\frac{1}{{T}_{2}}=\frac{4C{{{\gamma }^{2}\mu }_{0}^{2}N}_{A}}{135\pi {Dr}}{\left[L\left(a\right){\mu }_{{sp}}\right]}^{2}$$

3 $$L\left(a\right)={{\coth }}\frac{4\pi a}{{KT}}+\frac{{KT}}{4\pi a}$$

4 $$a=M{B}_{0}{r}^{3}$$

where *C* is the concentration of nanoparticle. *L*(*a*) is Langevin function. *μ*_*0*_, *γ*, *N*_*A*_, *D*, *K* and *T* are constants that represent the permeability of free space, the gyromagnetic ratio of the proton, Avogadro’s number, water diffusion coefficient, Boltzmann constant and absolute temperature, respectively. According to the Eqs. (2) to (4), T2 value is inversely proportional to the square of *μ*_*sp*_ and *L*(*a*), and the value of *L*(*a*) increases nonlinearly with the enhancement of *B*_*0*_, *M* and *r*. At the identical magnetic field strength, *M* and *μ*_*sp*_ of the contrast agents are the determining factors for *T*_2_. Consequently, compared with PMNPs, AFNP1 with smaller *M* and *μ*_*sp*_ provides a weaker T2-decaying effect^13,35^, leading to longer *T*_2_ and shorter *T*_*1*_ and thus a better contrasting effect at UHF MRI.

### Ultra-sensitive UHF MRI of the microscopic primary tumour

**Fig. 4:**
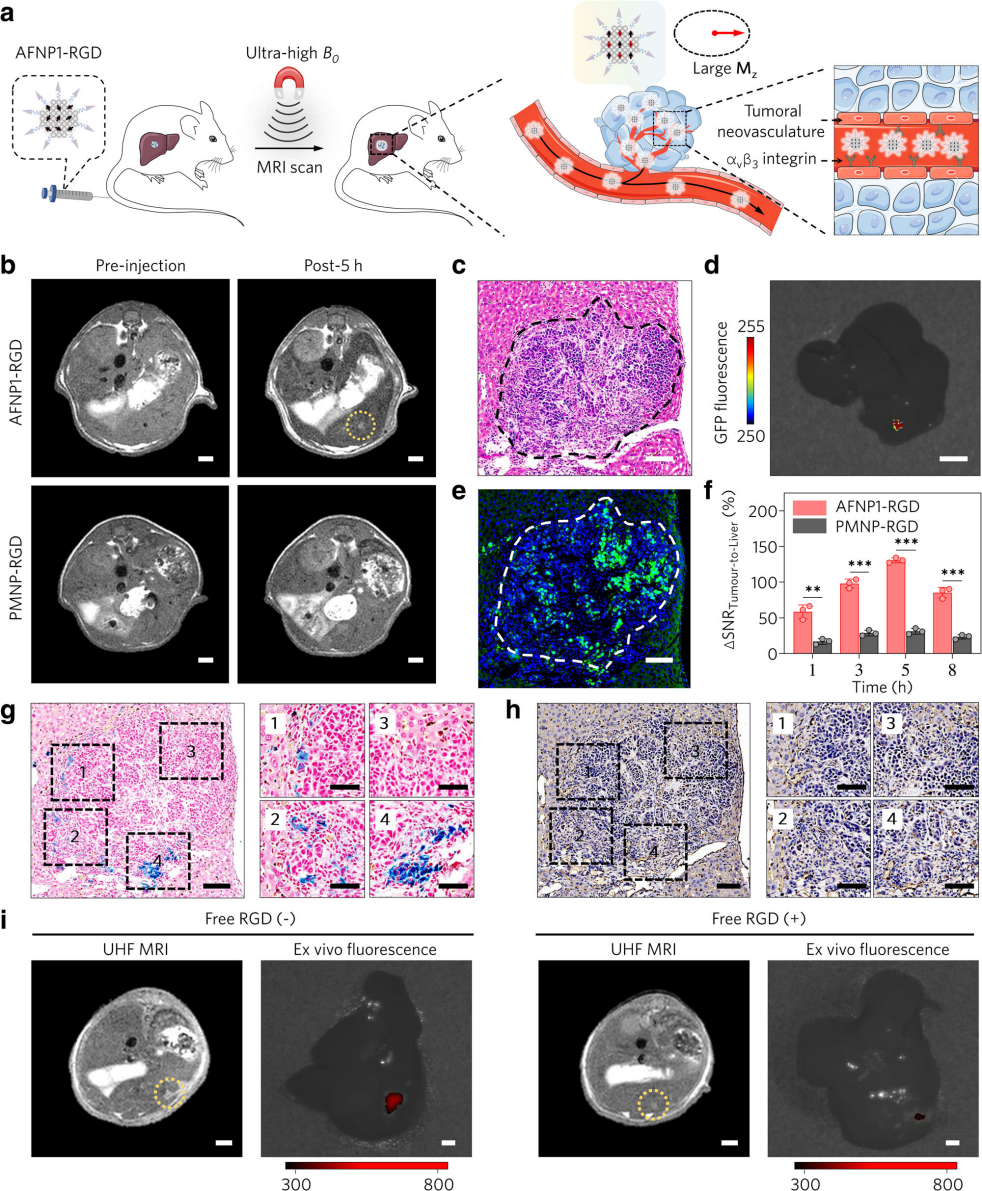
Ultra-sensitive UHF MRI of the microscopic primary tumour using AFNP1. **a** Schematic illustration of targeted UHF MRI of the hepatic microscopic primary tumour using AFNP1-RGD. **b** T1-weighted MR images of the mice-bearing microscopic primary hepatic tumour before and 5 h after the administration of AFNP1-RGD and PMNP-RGD (scale bar = 2 mm). Yellow circle represents the tumour region. **c** H&E staining of the microscopic primary tumour after the administration of AFNP1-RGD (scale bar = 100 μm). **d** Ex vivo GFP fluorescence image of the dissected liver tissue at 5 h after the injection of AFNP1-RGD (scale bar = 5 mm). **e** Fluorescence image of nuclei (blue) and GFP (green) in tumour (scale bar = 100 μm). **f** Quantitative analysis of ΔSNRs at the corresponding time points after i.v. injection of AFNP1-RGD and PMNP-RGD. *n* = 3 independent animals. Data are presented as mean ± SD. Data were compared using unpaired two-tailed Student’s *t* tests. ^**^P = 0.0024 (1 h), ^***^P = 0.000088 (3 h), ^***^P = 0.0000057 (5 h), ^***^P = 0.00017 (8 h). **g** Prussian blue staining of the liver section containing the primary tumour (scale bar =  100 μm) and magnified images of the annotated regions (scale bar = 50 μm). **h** CD31 staining of the microscopic primary hepatic tumour after the administration of AFNP1-RGD (scale bar = 100 μm) and magnified images of the annotated regions (scale bar = 50 μm). **i** The UHF MRI of primary hepatic tumour and ex vivo fluorescence imaging of the liver tissue at 5 h post single injection of RITC-labelled AFNP1-RGD or co-injection of RITC-labelled AFNP1-RGD and free RGD peptide (scale bar = 2 mm). Yellow circles represent the tumour regions. In **c**, **e**, **g**, **h**, experiments were repeated three times independently. Source data are provided as a Source Data file.

The high imaging performance of AFNP1 at UHF MRI prompted us to investigate their application for in vivo microscopic tumour diagnosis. The hypoxic microenvironment of microscopic tumours would stimulate the upregulation of integrin α_v_β_3_ in endothelial cells, thus promoting neoangiogenesis^44^. The cRGDyK that specifically bind to integrin α_v_β_3_^45^ were conjugated onto AFNP1 (AFNP1-RGD) and PMNP (PMNP-RGD) for targeted tumour imaging (Supplementary Figs. 6 and 7), and there is no obvious *r*_1_ and *r*_2_/*r*_1_ value change after cRGDyK modification (Supplementary Fig. 8), since the surface modification with cRGDyK does not change the magnetic core size, the surface chemical environment or the thickness of the hydrophilic coating layer^46^. AFNP1-RGD (*t*_1/2_ = 1.98 h) and PMNP-RGD (*t*_1/2_ = 1.79 h) with long blood circulation times (Supplementary Fig. 9) were intravenously (i.v.) injected into the orthotopic hepatic green fluorescent protein (GFP) labelled tumour-bearing mice for MRI (Fig. 4a). The microscopic primary tumour, as small as ~0.60 mm in size, is successfully detected and clearly distinguished from the normal liver tissue at 5 h post injection of AFNP1-RGD (Fig. 4b and Supplementary Fig. 10), which represents the lowest detection limit ever reported for primary hepatic tumours (Supplementary Table 2). The imaging performances of AFNP1-RGD and PMNP-RGD were also directly compared in the same tumour-bearing mouse that was sequentially administrated with these two RGD-conjugated nanoprobes (Supplementary Fig. 11). Moreover, the tumour size as determined by using AFNP1-RGD enhanced MRI (~0.60 mm) is nearly identical to that examined by hematoxylin and eosin (H&E) staining (~0.63 mm) and fluorescence imaging (~0.62 mm) (Fig. 4c–e), demonstrating the accurate delineation of tumour size can be achieved by AFNP1-RGD enhanced UHF MRI. The difference in ΔSNR value of the tumour tissue contrasted by AFNP1-RGD (~130.6%) is significantly higher than that contrasted by PMNP-RGD (~31.2%) (Fig. 4f). Prussian blue staining showed that the AFNP1-RGD accumulated around the edge of the tumour (Fig. 4g), which mostly colocalized with the tumour neovasculature (Fig. 4h). ICP-MS result (Supplementary Fig. 12) further confirmed the tumour accumulation of AFNP1-RGD. The tumour specificity of AFNP1-RGD was demonstrated by the competitive imaging studies in hepatic tumour-bearing mice with co-injection of free RGD peptide (Fig. 4i and Supplementary Figs. 13 and 14). Moreover, using a clinical 7-T MRI scanner equipped with a radiofrequency (RF) receiver coil for human use (Supplementary Fig. 15), AFNP1-RGD still enabled the detection of a small primary hepatic tumour with a size down to ~1 mm in rat, which holds great promise to translate into the ultra-sensitive clinical assessment of microscopic tumours.

### Ultra-sensitive UHF MRI of micrometastases

**Fig. 5:**
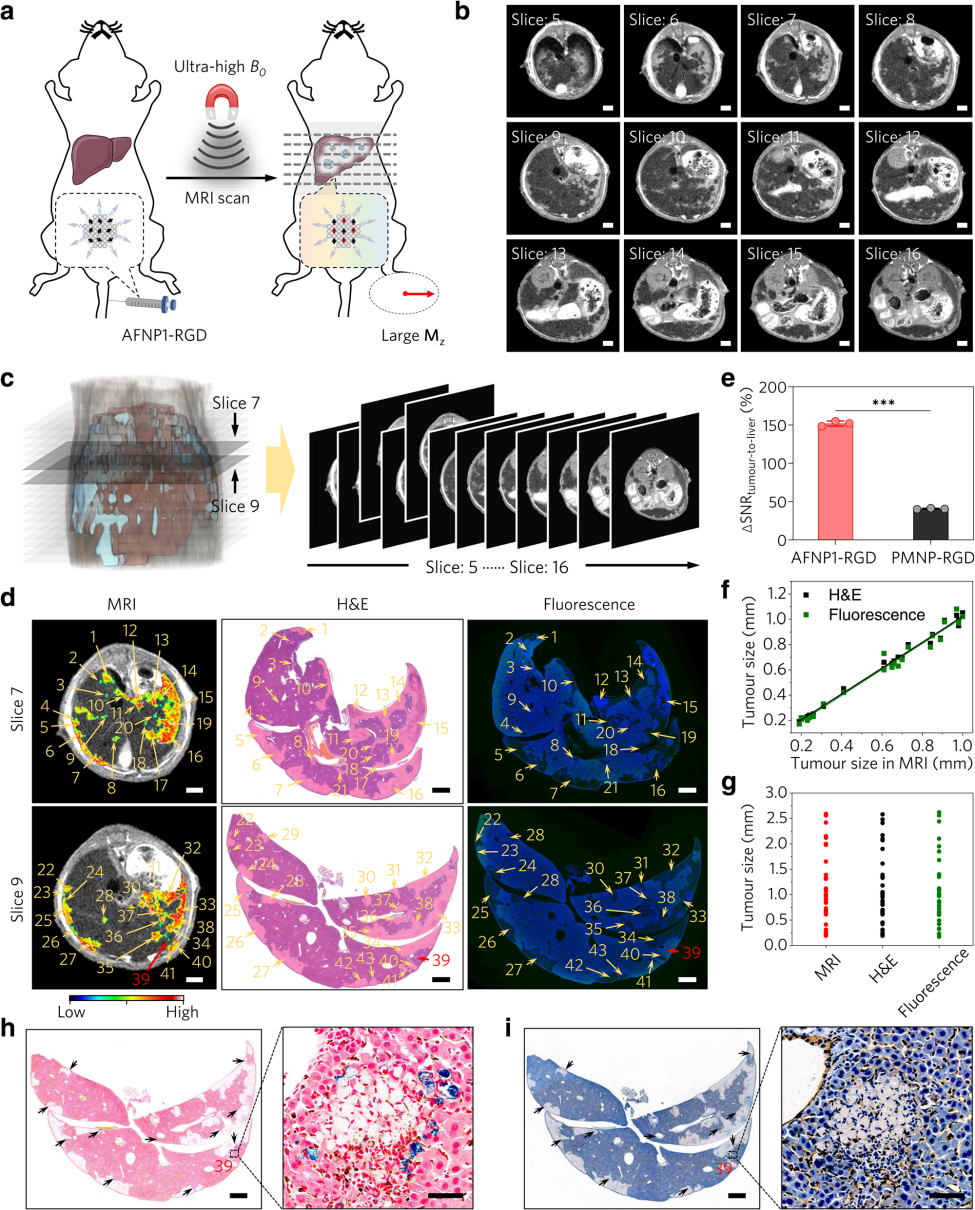
Ultra-sensitive UHF MRI of micrometastases using AFNP1. **a** Schematic illustration of targeted UHF MRI of hepatic micrometastases using AFNP1-RGD. **b** T1-weighted MR images in the transverse plane of mice-bearing hepatic micrometastases at 5 h post injection of AFNP1-RGD (scale bar = 2 mm). **c** 3D view constructed from AFNP1-RGD enhanced MRI of metastatic tumours (blue colour) in the liver (reddish-brown colour). **d** T1-weighted MR images, H&E-stained images and fluorescence images (nuclei in blue and GFP in green) of hepatic metastases-bearing mice at 5 h after the administration of AFNP1-RGD (scale bar = 2 mm for MRI; scale bar = 1000 μm for H&E-stained images and fluorescence images). Arrows and numbers indicate metastases, and the red arrow shows the micrometastasis with a size of ~0.20 mm. The same numbers indicate that the annotated metastases are matched according to their relative positions. **e** Quantification of ΔSNRs of MRI at 5 h after administration of AFNP1-RGD and PMNP-RGD. *n* = 3 independent animals. Data are presented as mean ± SD. Data were compared using unpaired two-tailed Student’s *t* tests. ^***^P = 0.00000058. **f** The sizes of micrometastases (<1 mm) detected by AFNP1-RGD enhanced MRI correlate well with those from H&E staining (*y* = 1.0228*x* + 0.0004; *R*^2^ = 0.989) and fluorescence imaging (*y* =  1.0284*x* − 0.0131; *R*^2^ = 0.977). **g** Sensitivity of AFNP1-RGD-enhanced MRI in detecting metastases (<5 mm), based on the sizes of 41 metastatic tumours analysed. **h** Prussian blue staining and **i** CD31 staining images of mice-bearing hepatic micrometastases at 5 h post injection of AFNP1-RGD (scale bar = 1000 μm; magnified images scale bar = 50 μm). In **d** (micrographs), **h**, **i**, experiments were repeated three times independently. Source data are provided as a Source Data file.

Metastasis is the most frequent cause of death for cancer patients^47^, the accurate and sensitive detection of metastatic tumours is highly valuable to patient stratification and prognosis. We further investigated the imaging performances of nanoprobes in mice models with hepatic micrometastases (Fig. 5a). At UHF MR scanning, metastatic tumours of different sizes are clearly visualised in both transverse plane (Fig. 5b) and three-dimensional (3D) reconstructed images of the liver at 5 h post i.v. injection of AFNP1-RGD (Fig. 5c). Even the micrometastases as small as 0.20 mm (as confirmed by both H&E staining and fluorescence images) can be illuminated by MRI (Fig. 5d), extending the detection limit of MRI probes^31^ (Supplementary Table 3). In contrast, almost no imaging enhancement can be observed by using PMNP-RGD (Supplementary Fig. 16). Moreover, with AFNP1-RGD as the imaging probe, the ΔSNR value of the metastatic tumour reaches ~152.0%, which is significantly higher than that of PMNP-RGD-enhanced MRI (~40.7%) (Fig. 5e). The sizes of metastatic tumours revealed in two random MRI scanning planes correlate well with those determined from H&E-stained (*R*^2^ = 0.989) or fluorescence images (*R*^2^ = 0.977) (Fig. 5d, f). Notably, this correlation reaches the highest level that can be achieved between H&E staining and contrast agent-based MRI^48^ (Supplementary Table 4), demonstrating the high accuracy and fidelity of AFNP1-RGD-enhanced UHF MRI of metastatic tumours. Dramatically, AFNP1-RGD-enhanced MRI is able to detect 90.2% of the <5 mm metastatic tumours that can be detected by both H&E staining and fluorescence imaging (Fig. 5g). To the best of our knowledge, such a near-histopathological level sensitivity has never been reached by any clinically available non-invasive imaging modalities (Supplementary Tables 3 and 5), demonstrating the superiority of AFNP1-RGD-enhanced UHF MRI. Prussian blue and CD31 staining confirm that the AFNP1-RGD is enriched in the angiogenic sites of metastases (Fig. 5h, i). Eventually, the AFNP1-RGD can be excreted from the body through renal clearance (Supplementary Fig. 17), and in vivo toxicological analysis including the serum chemistry, haematology test and histopathological examination demonstrate that AFNP1-RGD is highly biocompatible (Supplementary Fig. 18).

## Discussion

Non-invasive UHF MRI is capable of providing excellent imaging resolution to delineate anatomical details^17^, showing the potential to achieve the histopathological level imaging performance. However, the sensitivity of this technology is far from satisfactory due to the lack of imaging probes tailored for the ultra-high magnetic field. Strategies have been developed to enhance the sensitivity of imaging probes at the ultra-high magnetic field, e.g., metal chelates binding with macromolecules^19^, surface modification of the nanoparticular probes^49^. Nevertheless, most imaging probes reported thus far are paramagnetic or superparamagnetic species that can induce a strong T2-decaying effect at UHF MRI, leading to the slow T1 relaxation of water protons which impedes the contrast enhancement^13,26,35^. In this study, we are specifically interested in the artificial engineering of intrinsic magnetism of imaging probes that can influence the T1 relaxation of water protons. By controlling the magnetic properties of the probes including spin alignments, magnetic moments and magnetisation, we have systematically elucidated the relationship between UHF MRI performance and the magnetic properties of nanoprobes and demonstrated that antiferromagnetic nanoprobe with small magnetisation can effectively weaken the T2-decaying effect, substantially facilitating the recovery of T1 relaxation at UHF MRI. The optimised antiferromagnetic nanoprobe, AFNP1, possesses an ultralow magnetisation (0.23 emu g^−1^) and exhibits the ultrasmall *r*_2_/*r*_1_ value (~1.93) at 9 T MRI.

The ultimate goal of cancer molecular imaging is to non-invasively detect microscopic primary tumours or micrometastases at their early stage with high sensitivity and accuracy. In our study, UHF MRI-tailored AFNP1-RGD confers strong signal enhancement for effective identification of microscopic primary tumours, which still holds true when a clinical 7 T MRI scanner equipped with larger RF receiver coils is applied, allowing the detection of a primary hepatic tumour with a size down to ~1 mm. This result indicates the great potential of AFNP1-RGD enhanced UHF MRI in early diagnosis of primary hepatic tumours far smaller than stage T1a (solitary tumour size ≤2 cm in American Joint Committee on Cancer (AJCC) tumour/node/metastasis (TNM) classification system)^50^. More importantly, upon using the mice dedicated hardware, AFNP1-RGD-enhanced UHF MRI allows visualisation of the micrometastases in mice down to 0.20 mm with an ultra-high sensitivity (90.2% of H&E staining or fluorescence imaging), extending the detection limit of current clinical imaging modalities for non-invasive, high-resolution and accurate detection of microscopic primary or metastatic tumours. Consequently, the combination of high anatomical resolution of UHF MRI and UHF-tailored antiferromagnetic nanoprobes successfully enabled highly-sensitive non-invasive detection of microscopic primary tumours and micrometastasis at the histopathological level accuracy. In addition, AFNP1-RGD with a hydrodynamic size larger than 10 nm can be cleared from the body through the renal excretion, which may be ascribed to alternative clearance pathways other than glomerular filtration that allows removal of particles larger than the filtration threshhold^51–53^, e.g., active transcytosis of peritubular endothelium and tubular epithelial cells^51^.

To conclude, a rationally designed high-performance antiferromagnetic nanoprobe may open an avenue for developing next-generation ultra-sensitive MRI contrast agents, which are crucial to the visualisation of many previously undetectable biological lesions at UHF MRI. Ultimately, the conceptual advancements of the nanoprobe-enhanced UHF MRI may pave the way for future biomedical imaging, e.g., upon surface modification with appropriate targeting ligands, the ultra-sensitive UHF MRI detection of various types of tumours or other biological lesions at histopathological level, monitoring disease progression at very early stages, and accurately guiding remotely controlled therapies.

## Methods

### Chemicals and materials

All chemicals and materials used were commercially available and applied without further purification. Iron (III) acetylacetonate (98%), oleic acid (90%), dibenzyl ether were purchased from Sigma-Aldrich Co. (St. Louis, MO, USA). Platinum (II) acetylacetonate (97%), oleylamine (80–90%), oleyl alcohol, diphenyl ether, N-(3-dimethylaminopropyl)-N′-ethylcarbodiimide hydrochloride, N-hydroxysuccinimide, N, N’-dicyclohexylcarbodiimide, dopamine hydrochloride, 2-(N-morpholino) ethanesulfonic acid (MES) buffer and rhodamine B isothiocyanate (RITC) were purchased from Aladdin Industrial Inc. (Shanghai, China). Ethanol, hexane, diethyl ether, chloroform, dimethylformamide and sodium carbonate were purchased from Sinopharm Chemical Reagent Co., Ltd. (Shanghai, China). α,ω-dicarboxyl poly(ethylene glycol) (molecular weight = 2000) was acquired from Ponsure Biological Co. (Shanghai, China). Cyclic arginyl–glycyl-aspartic acid (RGD) peptide (cRGDyK) was obtained from GL Biochem (Shanghai) Ltd. Foetal bovine serum (FBS) was purchased from Sijiqing Biologic Co., Ltd. (Hangzhou, China). Dulbecco’s modified eagle medium (DMEM) and trypsin-EDTA solution (0.5% trypsin, 5.3 mM EDTA tetrasodium) were purchased from Jinuo Biomedical Technology Co., Ltd. (Hangzhou, China). Distilled water was prepared by Milli-Q (Millipore, USA).

### Characterisation

TEM images were acquired on a transmission electron microscope (Hitachi HT7700, Japan). HRTEM images were obtained on a transmission electron microscope (FEI Tecnai G2 F20 S-TWIN, U.S.A). Dynamic light scattering (DLS) measurements were conducted on a Nano ZS instrument (Malvern, UK). XRD patterns were obtained by using an X-ray diffractometer (PANalytical B.V. X-pert Powder, the Netherlands). Magnetic measurements were performed by a superconducting quantum interference device (SQUID) magnetometer (Cryogenic J3426, UK). Fourier-transform infrared spectra (FT-IR) were obtained by an infrared spectrophotometer (JASCO FI/IR-4100, Japan). XPS analysis was acquired via an XPS system (Thermo Scientific ESCALAB 250 Xi, UK). MRI images were obtained by a 9-T MRI scanner (Timemedical 9 T/110, USA). The amounts of Fe in samples were measured by inductively coupled plasma-mass spectrometry (ICP-MS, PerkinElmer NexION 300X, USA). The fluorescence images were acquired by the VISQUE InVivo Elite imaging system (Vieworks, Korea).

### Synthesis of AFNPs

For the synthesis of AFNP1, 40 mg of platinum (II) acetylacetonate and 35 mg of iron (III) acetylacetonate were mixed with 4 ml of benzyl ether at room temperature. The mixture was heated to 120 °C under an inert atmosphere. Once the solution reached 120 °C, 0.25 mmol of oleic acid and oleylamine as mixed surfactants were added. The solution was then heated to 210 °C and kept at that temperature for 30 min. The black solution was refluxed at 300 °C for 30 min then cooled down to room temperature, and excess ethanol was added to precipitate the nanoparticles. After centrifugation, the nanoparticles were dispersed in hexane. The different-sized AFNPs (AFNP2, AFNP3, AFNP4, AFNP5) were prepared following the same procedure but using a different amount of oleic acid and oleylamine of 0.5, 0.8, 1, 1.2 mmol, respectively.

### Preparation of W-AFNPs

In total, 60 mg of α,ω-dicarboxyl poly (ethylene glycol) (molecular weight = 2000), 6 mg of N-Hydroxysuccinimide, 9 mg of N, N’-dicyclohexylcarbodiimide, 4.2 mg of dopamine hydrochloride and 30 mg of sodium carbonate were dissolved in a mixture solvent containing 6 ml of chloroform and 3 ml of dimethylformamide. The mixed solution was stirred for 2 h and 15 mg of AFNPs were then added, followed by continuously stirring overnight at room temperature. After the reaction, the nanoparticles were precipitated by adding hexane, and then dispersed in 10 ml of distilled water. To remove excess surfactants and salts, the nanoparticle solution was dialysed against distilled water for 48 h, and followed by filtration using a 0.22-μm syringe filter to obtain W-AFNPs.

### Targeting modification of AFNP1

The immobilisation of cRGDyK on W-AFNP1 was achieved by an amide condensation reaction. N-(3-dimethylaminopropyl)-N’-ethylcarbodiimide hydrochloride (250 mg), N-hydroxysuccinimide (300 mg) and W-AFNP1 (0.2 mg Fe ml^−1^) were dissolved in the 2-(N-morpholino) ethanesulfonic acid (MES) buffer (10 ml) at room temperature. Subsequently, 5 mg of cRGDyK was added into the mixture, then the mixture was stirred overnight. After the reaction, the products were purified by dialyzing against distilled water for 48 h, and followed by filtration using a 0.22-μm syringe filter to obtain AFNP1-RGD that were stored at 4 °C for further studies.

### Synthesis of paramagnetic nanoparticle probe (PMNP)

In this study, small-sized iron oxide nanoparticles were fabricated as a typical PMNP. These nanoparticles were synthesised using the following method. In detail, 1.8 g of iron-oleate complex and 3.22 g of oleyl alcohol were dissolved in 10 g of diphenyl ether at room temperature. The mixture solution was heated to 200 °C at a constant heating rate of 10 °C min^−1^, and the reaction was kept at this temperature for 30 min. After the reaction, the resulting solution containing the nanoparticles was cooled down to room temperature, and excess acetone was added to the solution to precipitate the nanoparticles. The nanoparticles were separated by centrifugation.

### Preparation of water-dispersible PMNPs (W-PMNPs)

The PMNPs were easily transformed into the aqueous medium by a simple and effective ligand exchange reaction with the carboxylated poly (ethylene glycol). 60 mg of α,ω-dicarboxyl poly (ethylene glycol), 6 mg of N-hydroxysuccinimide, 9 mg of N, N’-dicyclohexylcarbodiimide, 4.2 mg of dopamine hydrochloride and 30 mg of sodium carbonate were dissolved in a mixture solvent containing 6 ml of chloroform and 3 ml of dimethylformamide. This mixed solution was stirred for 2 h at room temperature and 15 mg of PMNPs were then added, followed by continuously stirred overnight at room temperature. After the reaction, the nanoparticles were precipitated by adding hexane, followed by centrifugation. The nanoparticles were then dispersed in distilled water and purified by dialyzing against distilled water for 48 h. Any precipitations were removed using a 0.22-μm syringe filter to obtain W-PMNPs.

### Targeting modification of PMNPs

The immobilisation of cyclic arginyl–glycyl-aspartic acid (RGD) peptide (cRGDyK) on W-PMNPs was obtained by an amide condensation reaction. N-(3-dimethylaminopropyl)-N’-ethylcarbodiimide hydrochloride (250 mg), N-Hydroxysuccinimide (300 mg) and W-PMNP (2 mg Fe ml^−1^) were dissolved in the MES buffer at room temperature. Subsequently, cRGDyK (5 mg) was added to the mixture and then the mixture was continuously stirred overnight. After the reaction, the resulting product was purified by dialyzing against distilled water for 48 h. Any precipitation was removed by a 0.22-μm syringe filter, and the cRGDyK conjugated PMNP (PMNP-RGD) was acquired then stored at 4 °C for further studies.

### Measurement of released Pt and Fe ions from AFNP1-RGD

The Fe and Pt ions release were analysed by using dialysis tubes at 37 °C. AFNP1-RGD (456.2 μg Fe ml^−1^ and 3588.4 μg Pt ml^−1^) in 1 ml of phosphate-buffered saline (PBS) were placed in dialysis tubes and dialysed against 10 ml of PBS (pH = 7.4). The amount of released Fe and Pt ions were then quantified via ICP-MS.

### MRI relaxation properties of nanoprobes

MRI relaxivities of W-AFNPs and W-PMNPs were measured using an UHF 9-T MR scanner (Timemedical, 9 T/110). Inversion recovery (IR)–spin echo (SE) sequence was used to measure *T*_1_ (*T*_*R*_ incorporate the IR delay, *T*_*R*_ = 8000 ms, *T*_*E*_ = 9.18 ms, and inversion time (*TI*) = 50–5000 ms). *T*_1_ was mapped voxel-wise by fitting inversion recovery signal using the trust-region-reflective non-linear least-square fitting algorithms written in MATLAB R2017b. The inversion recovery *T*_1_ value is fitted by the equation, *M* = *M*_*0*_ (1–2*exp (−*TI*/*T*_*1*_)), where *M* is the longitudinal magnetisation of the water proton. For the calculation of *r*_1_ value, *R*_1_ = 1/*T*_1_ and *R*_1_ = *R*_10_ + [CA]*r_1_, where [CA] is the concentration of the contrast agent, *r*_1_ is the T1 relaxivity of the contrast agent. In addition, multi-slice multi-echo (MSME) sequence was used to measure *T*_2_ (*T*_*R*_ = 5000 ms, *T*_*E*_ = 11.7–93.9 ms, echo train length (ETL) = 8).

### Cell viability assay

RAW 264.7 cells (murine macrophage cell line; American Type Culture Collection (ATCC), Manassas, VA) were incubated in DMEM supplemented with 10% FBS and 1% penicillin/streptomycin at 37 °C in a humidified atmosphere of 5% CO_2_. To determine the cytotoxicity of AFNP1-RGD, the cells were plated in 96-well plates at a density of 1 × 10^4^ cells/well. After incubation for 24 h, the cells were exposed to 0, 1, 2, 5, 9, 19, 38, 150 and 300 μg Fe ml^−1^ of AFNP1-RGD. Cytotoxicity was assessed with a standard Cell Counting Kit-8 (CCK-8) assay after 24 h treatment.

### Preparation of RITC-labelled RGD targeted nanoprobes

In total, 5 ml of AFNP1-RGD (1 mg Fe ml^−1^) and 1 ml RITC (5 mg ml^−1^) were mixed for 24 h and dialysed against water for 48 h to obtain RITC-labelled AFNP1-RGD. PMNP-RGD and RITC were mixed to prepare RITC-labelled PMNP-RGD by the same procedure as that for the RITC-labelled AFNP1-RGD.

### Animal experiments

All animal experiments were performed in accordance with the guidelines of the Institutional Animal Care and Use Committee (IACUC) of Zhejiang University. All procedures were approved by the IACUC of Zhejiang University. BALB/c nude mice (male, 4–5 weeks), BALB/c mice (male, 4–6 weeks) and Wistar rat (male, 150–200 g) were purchased from Shanghai SLAC Laboratory Animal Co., Ltd. and were housed in open-top caging in a 14–10 h light–dark cycle and maintained a room temperature of 25 °C with 40–60% humidity.

### In vivo UHF MRI

Hepatic microscopic primary tumour models were established using the following method. Briefly, 5 × 10^5^ of green fluorescence protein (GFP)-expressing human liver cancer cells (Huh7-GFP-Luc; OBiO Technology (Shanghai) Corp., Ltd., Shanghai, China) were suspended in 20 µl of phosphate-buffered saline (PBS) and then injected into the left lobe of the liver of the BALB/c nude mice (4–5 weeks). One week later, the mice-bearing primary hepatic tumours (<1 mm) were divided into two groups (*n* = 3 for each group) for further MRI studies using a 9-T MRI scanner. The mice were subjected to inhalation anaesthesia using a 1.5% isoflurane-oxygen mixture, followed by i.v. injection of AFNP1-RGD or PMNP-RGD (5 mg Fe kg^−1^) for MRI. Images were obtained using a SE sequence (flip angle = 90°, *T*_*R*_ = 350 ms, *T*_*E*_  = 7.4 ms, in-plane field of view (FOV) = 40 × 40 mm^2^, matrix = 256 × 256, slice thickness/gap = 1.0 mm/0.1 mm, averages = 5, slices = 20, ETL = 1. Resolution: 0.16 × 0.16 × 1 mm. Scan duration: 7 min 52 s. Pixel intensity in each image dataset is fixed within the same range of 0–4095, with the maximum intensity set as 4095). The hepatic metastasis models were established in BALB/c nude mice (4–5 weeks) via intrasplenic injection of 100 μl PBS containing 1 × 10^5^ of Huh7-GFP-Luc cells. For in vivo MRI studies, the mice were i.v. injected with AFNP1-RGD or PMNP-RGD (5 mg Fe kg^−1^), then the same sequence and parameters used in primary tumour scanning were applied to acquire MR images (flip angle = 90°, *T*_*R*_ = 350 ms, *T*_*E*_ = 7.4 ms, FOV = 40 × 40 mm^2^, matrix = 256 × 256, slice thickness/gap = 1.0 mm/0.1 mm, averages = 5, slices = 20, ETL = 1. Resolution: 0.16 × 0.16 × 1 mm. Scan duration: 7 min 52 s. Pixel intensity in each image dataset is fixed within the same range of 0–4095, with the maximum intensity set as 4095).

The primary hepatic tumour-bearing rat models were established using the following method. In total, 5 × 10^6^ of Walker 256 cells (ATCC, Manassas, VA) were suspended in 50 μl of PBS and then injected into the left lobe of the liver in Wistar rat (150–200 g). Seven days later, the tumour-bearing rats were subjected to in vivo MRI studies using a clinical 7-T MRI scanner (Siemens Healthcare, Germany). The images were obtained by 3D flash sequence: Flip angle = 5°, *T*_*R*_ = 5.0 ms, *T*_*E*_ = 2.0 ms, FOV = 50 × 64 mm^2^, matrix = 150 × 192, slice thickness/gap = 1.0 mm/0.1 mm, averages = 5, slices = 32, ETL = 1. Resolution: 0.33 × 0.33 × 1 mm. Scan duration: 42.9 s. In addition, the imaging quality can be further improved by increasing the average number in clinically acceptable scan duration.

### Competitive imaging experiment

Twelve mice-bearing primary hepatic tumours were equally divided into two groups. The mice were subjected to inhalation anaesthesia using 1.5% isoflurane-oxygen mixture. For MRI, the mice were i.v. injected with RITC-labelled AFNP1-RGD/PMNP-RGD (5 mg Fe kg^−1^), or co-injected with RITC-labelled AFNP1-RGD/PMNP-RGD (5 mg Fe kg^−1^) and free peptide (200 mg kg^−1^). After in vivo MRI study, all the mice were sacrificed and the liver tissues were dissected. The fluorescence signals of the liver tissues were received in the “PE” channel of the VISQUE InVivo Elite imaging system.

### Ex vivo fluorescence imaging of hepatic tumour

After in vivo MRI of the primary hepatic tumour, the mice were sacrificed and the liver tissues were dissected. The GFP fluorescence signals of liver tissues were received in the “GFP” channel of the VISQUE InVivo Elite imaging system.

### MRI image analysis

The SNR value was calculated for each mouse to quantify the signal enhancement in the region of interest (ROI) from the following equations: SNR = SI_ROI,mean_/SI _tissue,mean_, where SI_mean_ stands for intensity in ROI or surrounding normal tissue from the MR images. The signal changes in ROI at different time points after the administration of nanoprobes were quantified using ΔSNR calculated according to the following equations: ΔSNR = (SNR_post_ −  SNR_pre_)/SNR_pre_ × 100%.

The greyscale images of hepatic metastases were converted to pseudo-colour images using ImageJ (Version 1.52a, National Institutes of Health, USA). According to the average colour range of primary hepatic tumours that were also validated by histopathology, the regions in the liver with a colour range of 130–250 were manually selected and converted to overlays, followed by merging with the greyscale images.

### Histopathological analysis

The AFNP1-RGD was i.v. injected into hepatic microscopic primary tumours or micrometastases bearing experimental mice. After 5 h, the mice were sacrificed, and the livers were harvested and fixed in 10% neutral buffered formalin. Then, the tissues were processed routinely into the paraffin and then sectioned. Lastly, the tissue sections were stained with hematoxylin & eosin (H&E), Prussian blue staining and CD31 staining, respectively.

### Blood half-life of nanoprobes

AFNP1-RGD or PMNP-RGD in 200 μl of PBS was i.v. injected into BALB/c mice at a dosage of 5 mg Fe kg^−1^. Blood samples were collected from the eye socket at 1 min, 15 min, 30 min, 1 h, 3 h and 8 h post injection. The plasma of each blood sample was obtained via centrifugation. Aqua regia (1 ml) was then added to each plasma sample and incubated for 24 h. The solutions were then diluted tenfold with 2% nitric acid. The determination of Fe content in blood was performed by using ICP-MS.

### Biodistribution analysis

Fe uptake in the main viscera of Huh7 tumour-bearing mice was assessed before and after i.v. injection of AFNP1-RGD or PMNP-RGD at a dosage of 5 mg Fe kg^−1^, respectively. After sacrificing the mice, the tissues were removed and weighed. In total, 1 ml of aqua regia was added to each sample and incubated for 24 h. The solutions were then diluted tenfold with 2% nitric acid. The determination of Fe content in the tissues was performed by using ICP-MS.

### In vivo toxicity evaluation

Healthy male balb/c mice were i.v. injected with AFNP1-RGD (5 mg Fe kg^−1^) or saline (control group). After 15 days, the mice were sacrificed and main viscera were collected. Heart, liver, spleen, kidney and lung tissues were harvested and stained with H&E. Blood sample was acquired by eye socket and prepared for haematology test, and the serum was isolated from the blood sample. Liver function was evaluated by determining aspartate aminotransferase (AST) and albumin (ALB) levels of the serum samples. Kidney function was evaluated by determining blood urea nitrogen (BUN) and creatinine (CREA) levels of the serum samples. In addition, the in vivo toxicity evaluation of AFNP1-RGD at 1-, 15- and 90-day post injection was also performed with the same procedures.

### Statistical analyses

Data analysis was performed by using OriginPro (version 8.5.0 SR1), Microsoft Excel (version 16.0.13929.20206), GraphPad Prism (version 8.0.2), ImageJ (version 1.52a), MATLAB (version R2017b), 3DSlicer (version 4.11.0), RadiAnt DICOM Viewer (version 2020.2.3), CaseViewer (version 2.0), Gatan DigitalMicrograph (version 2.10.1282.0). All of the data were presented as the mean ± SD from a minimum of three independent experiments.

### Reporting summary

Further information on research design is available in the Nature Research Reporting Summary linked to this article.

## Supplementary information

Supplementary Information

Peer Review File

Reporting Summary

## Data Availability

The data are available within the article, supplementary information or available from the authors upon request. Source data are provided with this paper.

## Source data

Source Data

## References

[1] Glaser AK (2017). Light-sheet microscopy for slide-free nondestructive pathology of large clinical specimens. Nat. Biomed. Eng..

[2] Richard L (2017). Histopathology is ripe for automation. Nat. Biomed. Eng..

[3] Mittal S (2018). Simultaneous cancer and tumor microenvironment subtyping using confocal infrared microscopy for all-digital molecular histopathology. Proc. Natl Acad. Sci. USA.

[4] Greenbaum A (2014). Wide-field computational imaging of pathology slides using lens-free on-chip microscopy. Sci. Transi. Med..

[5] Liu C (2019). Low-cost thermophoretic profiling of extracellular-vesicle surface proteins for the early detection and classification of cancers. Nat. Biomed. Eng..

[6] Giedt RJ (2018). Single-cell barcode analysis provides a rapid readout of cellular signaling pathways in clinical specimens. Nat. Commun..

[7] Krebs MG (2014). Molecular analysis of circulating tumour cells-biology and biomarkers. Nat. Rev. Clin. Oncol..

[8] Shin TH, Choi Y, Kim S, Cheon J (2015). Recent advances in magnetic nanoparticle-based multi-modal imaging. Chem. Soc. Rev..

[9] Park S, Aalipour A, Vermesh O, Yu JH, Gambhir SS (2017). Towards clinically translatable in vivo nanodiagnostics. Nat. Rev. Mater..

[10] Kunjachan S, Ehling J, Storm G, Kiessling F, Lammers T (2014). Noninvasive imaging of nanomedicines and nanotheranostics: principles, progress, and prospects. Chem. Rev..

[11] Kircher MF, Willmann JK (2012). Molecular body imaging: MR imaging, CT, and US. part I. principles. Radiology.

[12] Smith BR, Gambhir SS (2017). Nanomaterials for in vivo imaging. Chem. Rev..

[13] Zhou Z, Yang L, Gao J, Chen X (2019). Structure-relaxivity relationships of magnetic nanoparticles for magnetic resonance imaging. Adv. Mater..

[14] Lee N, Hyeon T (2012). Designed synthesis of uniformly sized iron oxide nanoparticles for efficient magnetic resonance imaging contrast agents. Chem. Soc. Rev..

[15] Na HB, Song IC, Hyeon T (2009). Inorganic nanoparticles for MRI contrast agents. Adv. Mater..

[16] Nowogrodzki A (2018). The strongest scanners. Nature.

[17] Duan G, Zhao X, Anderson SW, Zhang X (2019). Boosting magnetic resonance imaging signal-to-noise ratio using magnetic metamaterials. Commun. Phys..

[18] Kraff O, Fischer A, Nagel AM, Monninghoff C, Ladd ME (2015). MRI at 7 tesla and above: demonstrated and potential capabilities. J. Magn. Reson. Imaging.

[19] Hu H (2017). Dysprosium-modified tobacco mosaic virus nanoparticles for ultra-high-field magnetic resonance and near-infrared fluorescence imaging of prostate cancer. ACS Nano.

[20] Rammohan N (2016). Nanodiamond-gadolinium (III) aggregates for tracking cancer growth in vivo at high field. Nano Lett..

[21] Wahsner J, Gale EM, Rodriguez-Rodriguez A, Caravan P (2019). Chemistry of MRI contrast agents: current challenges and new frontiers. Chem. Rev..

[22] Viswanathan S, Kovacs Z, Green KN, Ratnakar SJ, Sherry AD (2010). Alternatives to gadolinium-based metal chelates for magnetic resonance imaging. Chem. Rev..

[23] Li H, Meade TJ (2019). Molecular magnetic resonance imaging with Gd (III)-based contrast agents: challenges and key advances. J. Am. Chem. Soc..

[24] Kim KS, Park W, Na K (2015). Gadolinium-chelate nanoparticle entrapped human mesenchymal stem cell via photochemical internalization for cancer diagnosis. Biomaterials.

[25] Kim D, Kim J, Park YI, Lee N, Hyeon T (2018). Recent development of inorganic nanoparticles for biomedical imaging. ACS Cent. Sci..

[26] Lee SH, Kim BH, Na HB, Hyeon T (2013). Paramagnetic inorganic nanoparticles as T_1_ MRI contrast agents. Wiley Interdiscip. Rev. Nanomed. Nanobiotechnol..

[27] Kim T (2011). Mesoporous Silica-coated hollow manganese oxide nanoparticles as positive T1 contrast agents for labeling and MRI tracking of adipose-derived mesenchymal stem cells. J. Am. Chem. Soc..

[28] Kwon HJ (2018). Large-scale synthesis and medical applications of uniform-sized metal oxide nanoparticles. Adv. Mater..

[29] Bai C (2018). Time‐dependent T1-T2 switchable magnetic resonance imaging realized by c(RGDyK) modified ultrasmall Fe_3_O_4_ nanoprobes. Adv. Funct. Mater..

[30] Zhao Y (2016). Bioengineered magnetoferritin nanoprobes for single-dose nuclear-magnetic resonance tumor imaging. ACS Nano.

[31] Li Y (2019). A bioinspired nanoprobe with multilevel responsive T1-weighted MR signal-amplification illuminates ultrasmall metastases. Adv. Mater..

[32] Lee J (2016). Targeted multimodal nano-reporters for pre-procedural MRI and intra-operative image-guidance. Biomaterials.

[33] Yuan Y (2019). Furin-mediated intracellular self-assembly of olsalazine nanoparticles for enhanced magnetic resonance imaging and tumour therapy. Nat. Mater..

[34] Miller JS (2011). Magnetically ordered molecule-based materials. Chem. Soc. Rev..

[35] Zhou Z (2017). T1-T2 dual-modal magnetic resonance imaging: from molecular basis to contrast agents. ACS Nano.

[36] Yu Y (2014). Monodisperse MPt (M = Fe, Co, Ni, Cu, Zn) nanoparticles prepared from a facile oleylamine reduction of metal salts. Nano Lett..

[37] Kim BH (2011). Large-scale synthesis of uniform and extremely small-sized iron oxide nanoparticles for high-Resolution T1 magnetic resonance imaging contrast agents. J. Am. Chem. Soc..

[38] Hong R, Fisher NO, Emrick T, Rotello VM (2005). Surface PEGylation and ligand exchange chemistry of FePt nanoparticles for biological applications. Chem. Mater..

[39] Shen L (2013). Room-temperature ferromagnetism in ZnO-encapsulated 1.9 nm FePt_3_ nanoparticle–composite thin films with giant interfacial anisotropy. Adv. Mater..

[40] Heitsch AT, Lee DC, Korgel BA (2010). Antiferromagnetic single domain L1_2_ FePt_3_ nanocrystals. J. Phys. Chem. C..

[41] Nemec P, Fiebig M, Kampfrath T, Kimel AV (2018). Antiferromagnetic opto-spintronics. Nat. Phys..

[42] Zeng J (2014). Anchoring group effects of surface ligands on magnetic properties of Fe_3_O_4_ nanoparticles: towards high performance MRI contrast agents. Adv. Mater..

[43] Laurent S (2008). Magnetic iron oxide nanoparticles: synthesis, stabilization, vectorization, physicochemical characterizations, and biological applications. Chem. Rev..

[44] Weis SM, Cheresh DA (2011). Tumor angiogenesis: molecular pathways and therapeutic targets. Nat. Med..

[45] Sun X (2017). Peptide-based imaging agents for cancer detection. Adv. Drug Deliv. Rev..

[46] Zhang W (2018). Surface impact on nanoparticle-based magnetic resonance imaging contrast agents. Theranostics.

[47] Steeg PS (2016). Targeting metastasis. Nat. Rev. Cancer.

[48] Tan S (2020). Chemokine receptor 4 targeted protein MRI contrast agent for early detection of liver metastases. Sci. Adv..

[49] Ni D, Bu W, Ehlerding EB, Cai W, Shi J (2017). Engineering of inorganic nanoparticles as magnetic resonance imaging contrast agents. Chem. Soc. Rev..

[50] Kamarajah SK, Frankel TL, Sonnenday C, Cho CS, Nathan H (2018). Critical evaluation of the American joint commission on cancer (AJCC) 8th edition staging system for patients with hepatocellular carcinoma (HCC): a surveillance, epidemiology, end results (SEER) analysis. J. Surg. Oncol..

[51] Naumenko V (2019). Intravital microscopy reveals a novel mechanism of nanoparticles excretion in kidney. J. Control. Release.

[52] Cheng L (2017). Renal-clearable PEGylated porphyrin nanoparticles for image-guided photodynamic cancer therapy. Adv. Funct. Mater..

[53] Gomez-Vallejo V (2018). PEG-Copolymer-coated iron oxide nanoparticles that avoid the reticuloendothelial system and act as kidney MRI contrast agents. Nanoscale.

