# Supplementary material for: Artificially engineered antiferromagnetic nanoprobes for ultra-sensitive histopathological level magnetic resonance imaging
Source: Nat Commun. 2021 Jun 22;12:3840. doi: 10.1038/s41467-021-24055-2 (PMC8219830; doi:10.1038/s41467-021-24055-2)
Supplement: Supplementary file 1 — Supplementary Information [file 41467_2021_24055_MOESM1_ESM.pdf]

Artificially engineered antiferromagnetic nanoprobes for ultra-sensitive histopathological level magnetic resonance imaging

Liang *et al.*

Supplementary Figures:

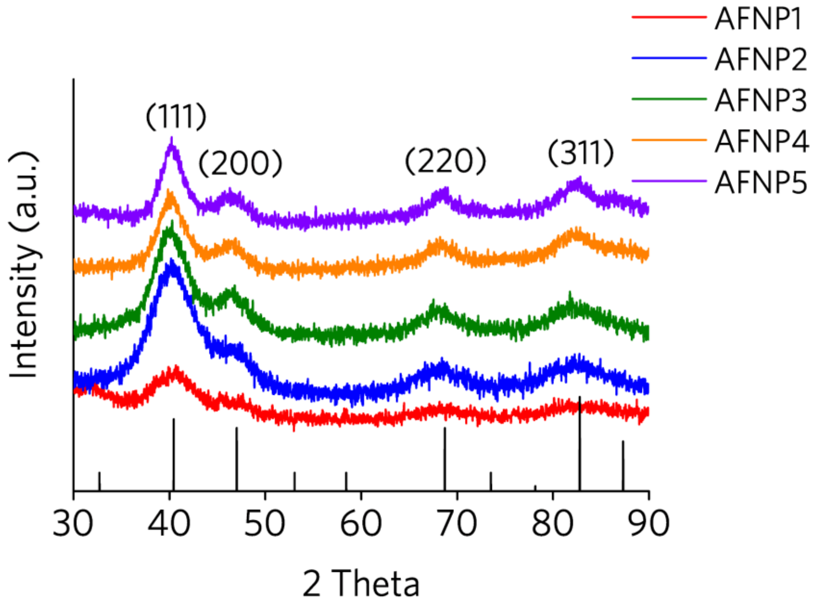

Supplementary Figure 1. X-ray powder diffraction (XRD) patterns of AFNPs. All the AFNPs reveal $FePt_3$ (JCPDS no. 29-0716) crystalline structures. Source data are provided as a Source Data file.

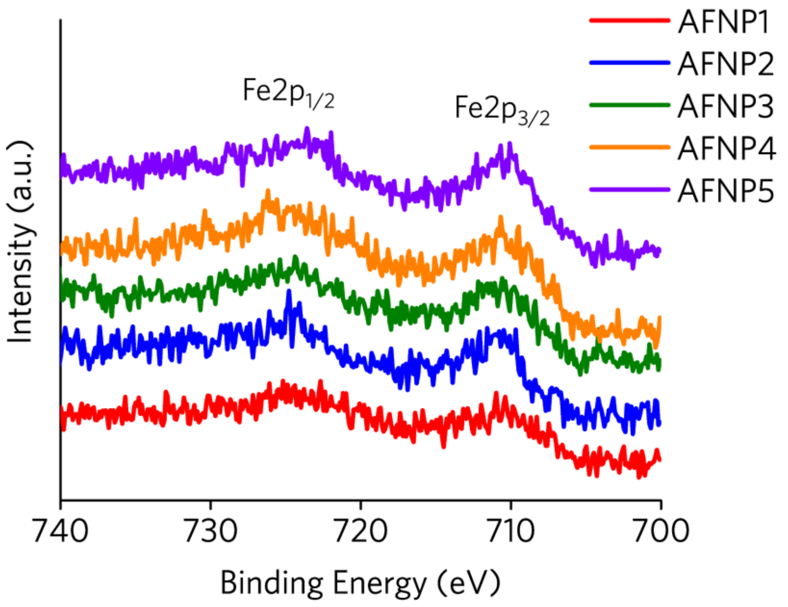

Supplementary Figure 2. X-ray photoelectron spectroscopy (XPS) analysis of AFNPs. The spectra of Fe2p contains main peaks at 710.98 eV and 725.48 eV corresponding to Fe $2p_{3/2}$ and $Fe2p_{1/2}$, respectively, which supports the presence of ferric (Fe^{3+}) ions on particle surface. Source data are provided as a Source Data file.

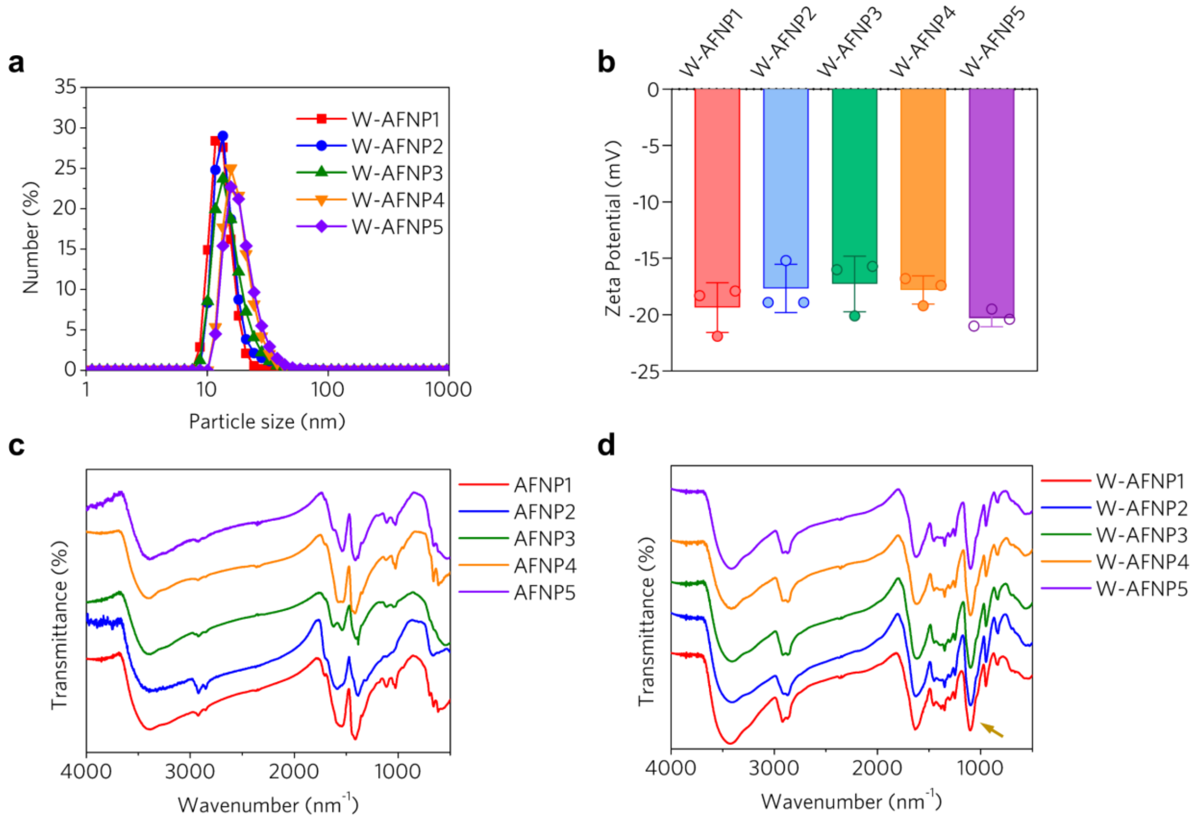

Supplementary Figure 3. Characterization of W-AFNPs. a, The hydrodynamic sizes and **b,** Zeta potentials of W-AFNP1, W-AFNP2, W-AFNP3, W-AFNP4, and W-AFNP5. $n = 3$ independent experiments. Data are presented as mean ± SD. Fourier-transform infrared spectroscopy (FT-IR) spectra of **c,** AFNP1, AFNP2, AFNP3, AFNP4, AFNP5 and **d,** W-AFNP1, W-AFNP2, W-AFNP3, W-AFNP4, W-AFNP5. Source data are provided as a Source Data file.

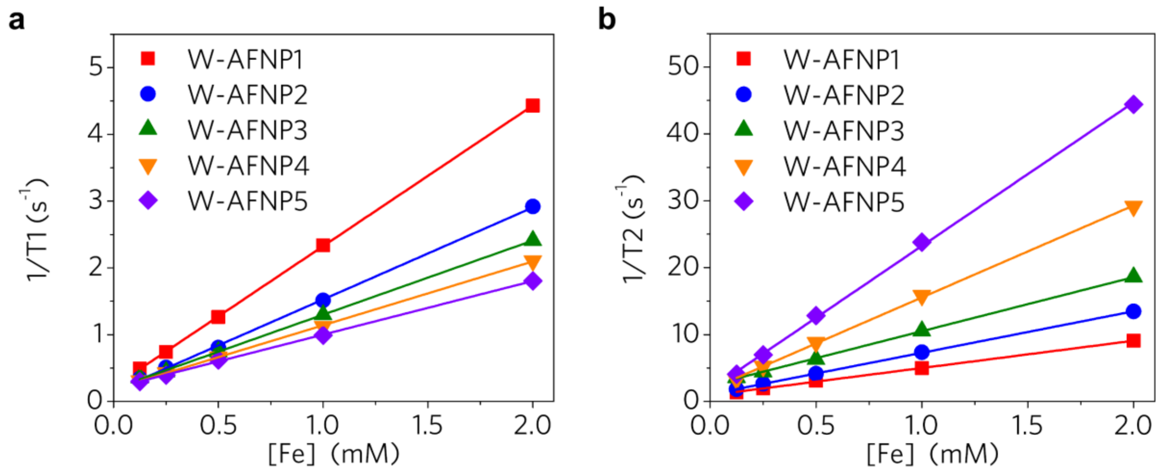

Supplementary Figure 4. Relaxivity of W-AFNPs. a, T1 relaxation rates (1/T1, s^{-1}) and **b,** T2 relaxation rates (1/T2, s^{-1}) plotted as a function of Fe concentrations (mM) for W-AFNP1, W-AFNP2, W-AFNP3, W-AFNP4, and W-AFNP5. Source data are provided as a Source Data file.

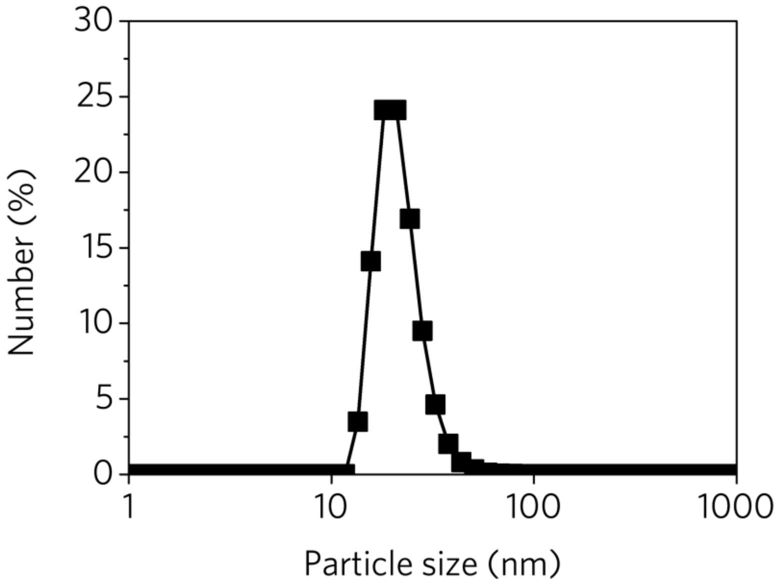

Supplementary Figure 5. Characterization of W-PMNP. The hydrodynamic size of W-PMNP. Source data are provided as a Source Data file.

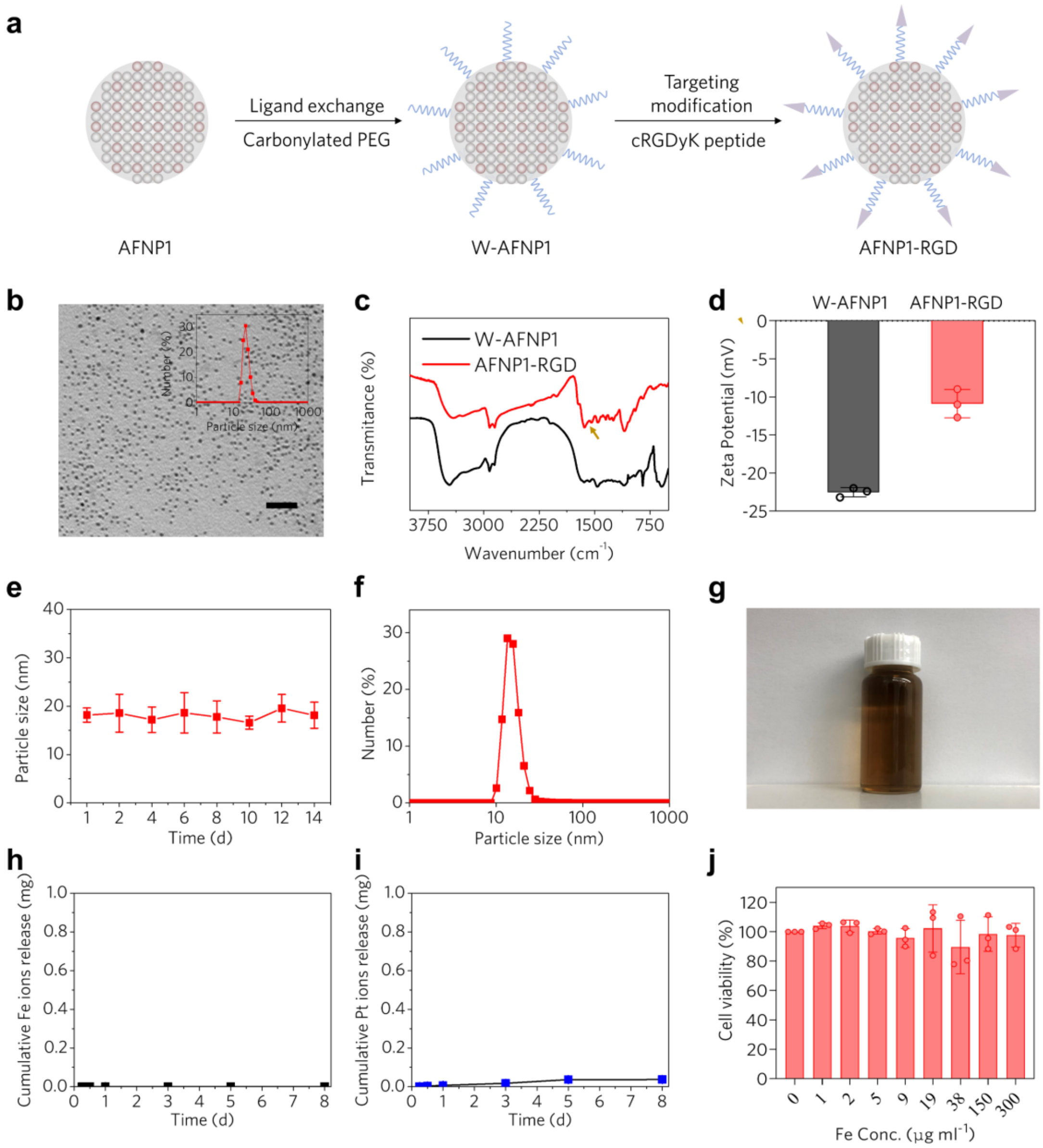

Supplementary Figure 6. Characterization of AFNP1-RGD. a, Schematic illustration of AFNP1-RGD preparation. **b,** The TEM image of AFNP1-RGD, scale bar = 20 nm. Inset: the hydrodynamic size of AFNP1-RGD. **c,** FT-IR spectra of W-AFNP1 and AFNP1-RGD. A new peak at 1641 cm^{-1} for the AFNP1-RGD (yellow arrow), suggesting that the C=N bonds have been formed between cRGDyK and W-AFNP1. **d,** Zeta potential of W-AFNP1 and AFNP1-RGD. *n* = 3 independent experiments. Data are presented as mean ± SD. **e,** The hydrodynamic sizes of AFNP1-RGD within 14 days in DMEM with FBS solution. *n* = 3 independent experiments. Data are presented as mean ± SD. **f,** The hydrodynamic size of AFNP1-RGD after 22 months placed in the PBS solution. **g,** Photograph of AFNP1-RGD dispersed in the PBS solution (0.1 mg ml^{-1}) placed with 23 months. **h,** Fe and **i,** Pt ions release from AFNP1-RGD in PBS at pH 7.4. After 8 days, only ~ 1.0 μg (~ 0.22 %) of Fe ions and ~ 37.2 μg (~ 1.04 %) of Pt ions were leached from AFNP1-RGD. *n* = 3 independent experiments. Data are presented as mean ± SD. **j,** In vitro cytotoxicity of the AFNP1-RGD to RAW 264.7 cells was evaluated by CCK-8 assay, indicating the biocompatibility of AFNP1-RGD. *n* = 3 independent experiments. Data are presented as mean ± SD. In **b** experiments were repeated three times independently. Source data are provided as a Source Data file.

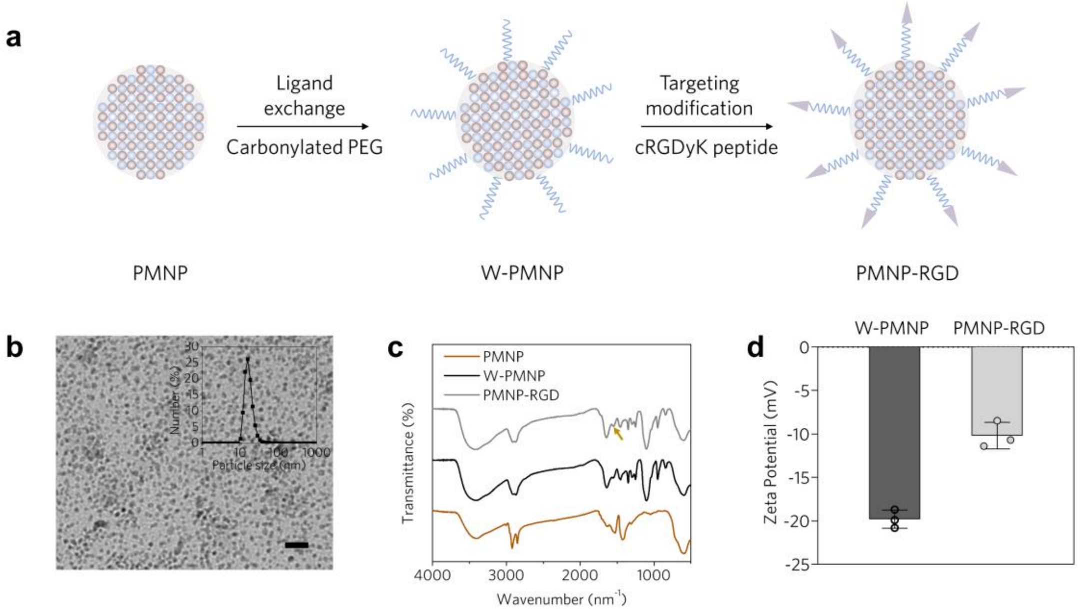

Supplementary Figure 7. Characterization of PMNP-RGD. a, Schematic illustration of PMNP-RGD preparation. **b,** The TEM image of PMNP-RGD, scale bar = 20 nm. Inset image: The hydrodynamic size of PMNP-RGD. **c,** FT-IR spectra of PMNP, W-PMNP and PMNP-RGD. **d,** Zeta potentials of W-PMNP and PMNP-RGD. n = 3 independent experiments. Data are presented as mean ± SD. In **b** experiments were repeated three times independently. Source data are provided as a Source Data file.

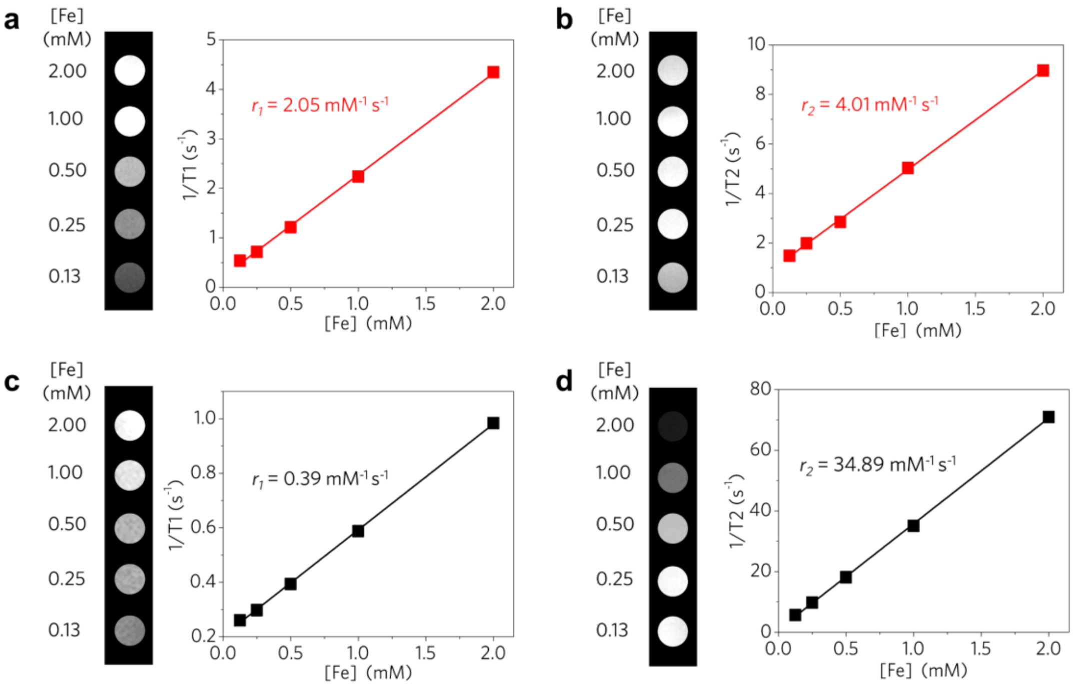

Supplementary Figure S8. Relaxivity of AFNP1-RGD and PMNP-RGD. a, T1-weighted images and T1 relaxation rates (1/T1, s^{-1}), and **b,** T2-weighted images and T2 relaxation rates (1/T2, s^{-1}) plotted as a function of Fe concentrations (mM) for AFNP1-RGD. **c,** T1-weighted images and T1 relaxation rates (1/T1, s^{-1}), and **d,** T2-weighted images and T2 relaxation rates (1/T2, s^{-1}) plotted as a function of Fe concentrations (mM) for PMNP-RGD. Source data are provided as a Source Data file.

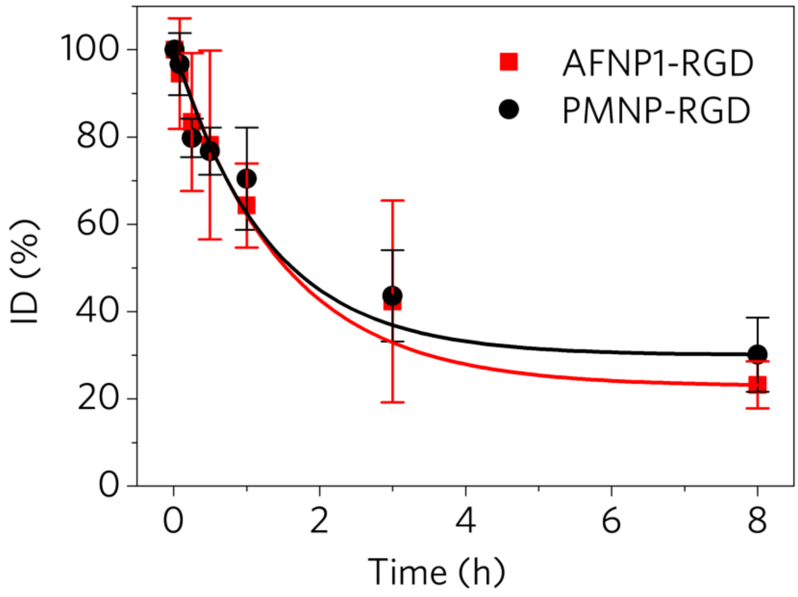

Supplementary Figure 9. In vivo pharmacokinetic curves of AFNP1-RGD and PMNP-RGD. Fe concentrations in the blood expressed as the percentage of the injected dose (% ID) of AFNP1-RGD and PMNP-RGD at different times after administration. The concentration is determined by measuring Fe content in blood via ICP-MS. $n = 3$ independent animals. Data are presented as mean ± SD. Source data are provided as a Source Data file.

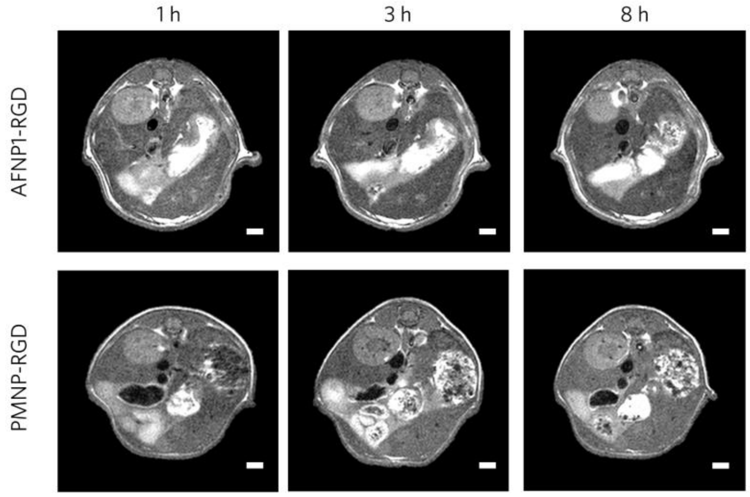

Supplementary Figure 10. UHF MRI of microscopic primary tumour using AFNP1-RGD and PMNP-RGD. T1-weighted MR images of mice bearing hepatic microscopic primary tumour at the time point of 1, 3, 8 h after the administration of AFNP1-RGD and PMNP-RGD (scale bar = 2 mm).

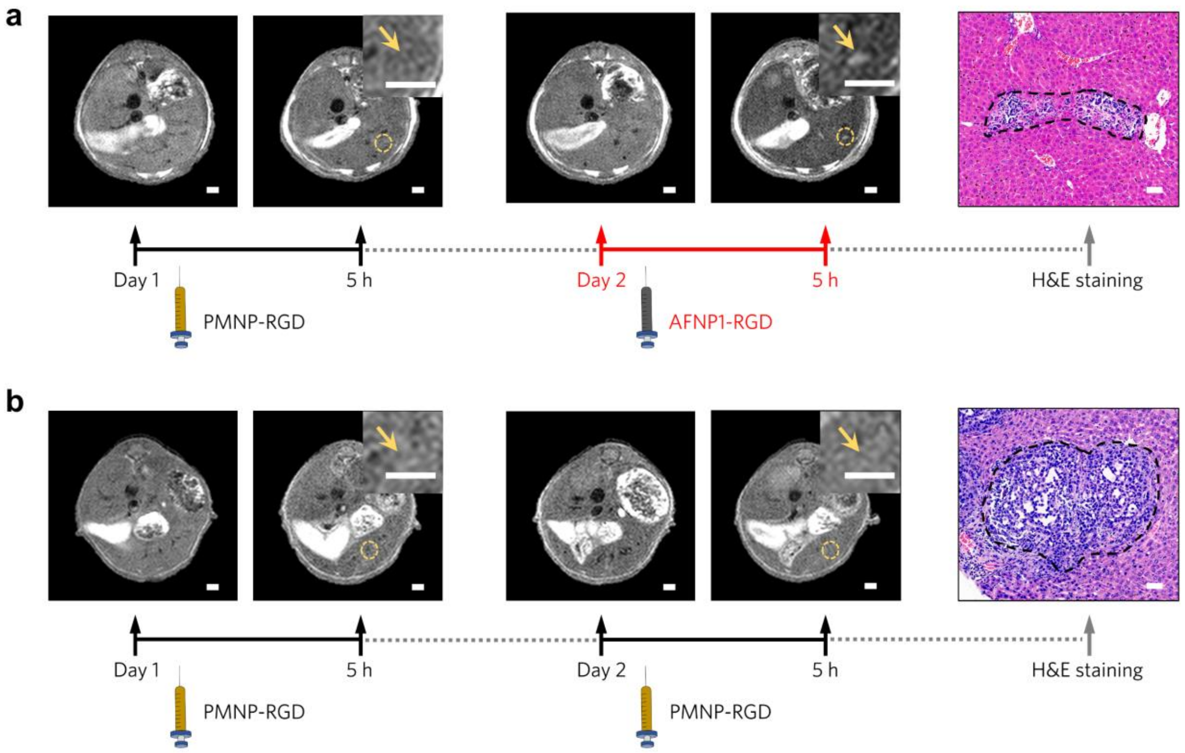

Supplementary Figure 11. The sequential administration of PMNP-RGD and AFNP1-RGD/PMNP-RGD in hepatic tumour-bearing mouse. PMNP-RGD (5 mg Fe kg^{-1}) was first i.v. injected into the mouse and then MRI was applied 5 h later. 24 h later, **a,** AFNP1-RGD (5 mg Fe kg^{-1}) or **b,** PMNP-RGD (5 mg Fe kg^{-1}) was injected into the same mouse and then MRI was also applied 5 h later (scale bar (T1-weighted images) = 2 mm; scale bar (H&E stained images) = 50 µm). In **a-b** (micrographs) experiments were repeated three times independently.

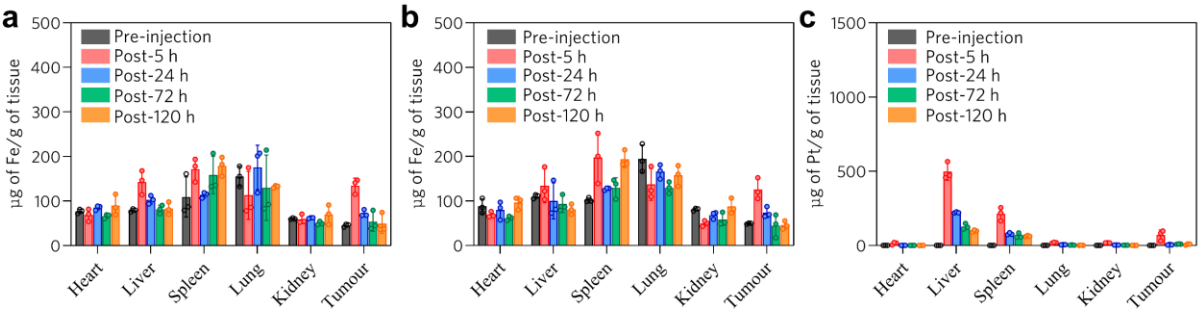

Supplementary Figure 12. In vivo biodistribution of AFNP1-RGD and PMNP-RGD. Tissue distributions of Fe before and after i.v. injection of **a,** AFNP1-RGD and **b,** PMNP-RGD into Huh7 tumour-bearing mice. *n* = 3 independent animals. Data are presented as mean ± SD. **c,** Tissue distributions of Pt before and after i.v. injection of AFNP1-RGD into Huh7 tumour-bearing mice. *n* = 3 independent animals. Data are presented as mean ± SD. Source data are provided as a Source Data file.

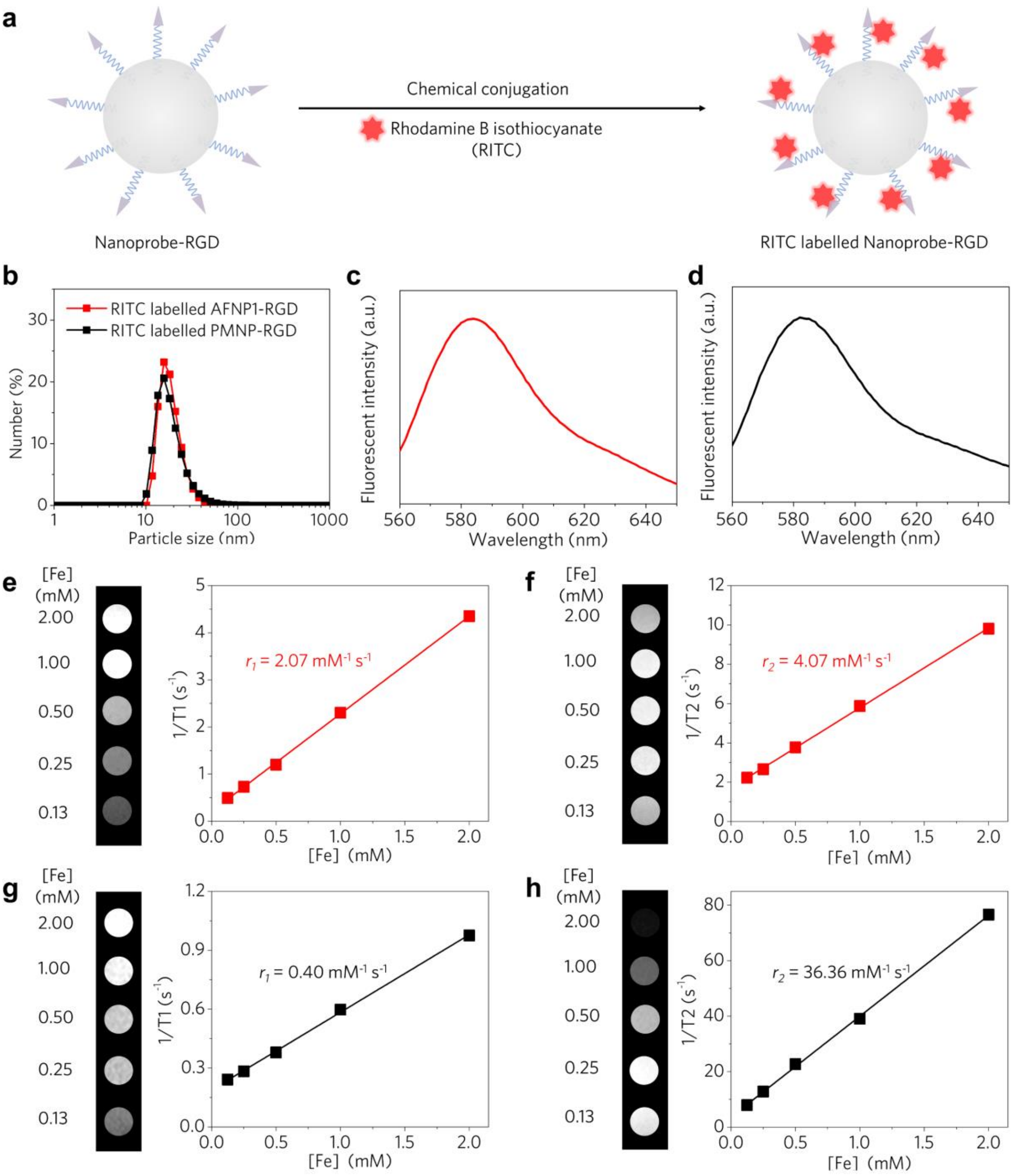

Supplementary Figure 13. Characterizations of RITC labelled AFNP1-RGD and PMNP-RGD. a, Schematic illustration for the labelling of RGD conjugated nanoprobes with RITC. **b,** The hydrodynamic size of RGD conjugated nanoprobes. **c,d,** Fluorescence spectra of RITC labelled (**c**) AFNP1-RGD and (**d**) PMNP-RGD, respectively. **e,** T1-weighted images and T1 relaxation rates (1/T1, s^{-1}) plotted against Fe concentrations (mM) for RITC labelled AFNP1-RGD. **f,** T2-weighted images and T2 relaxation rates (1/T2, s^{-1}) plotted against Fe concentrations (mM) for RITC labelled AFNP1-RGD. **g,** T1-weighted images and T1 relaxation rates (1/T1, s^{-1}) plotted against Fe concentrations (mM) for RITC labelled PMNP-RGD. **h,** T2-weighted images and T2 relaxation rates (1/T2, s^{-1}) plotted against Fe concentrations (mM) for RITC labelled PMNP-RGD. Source data are provided as a Source Data file.

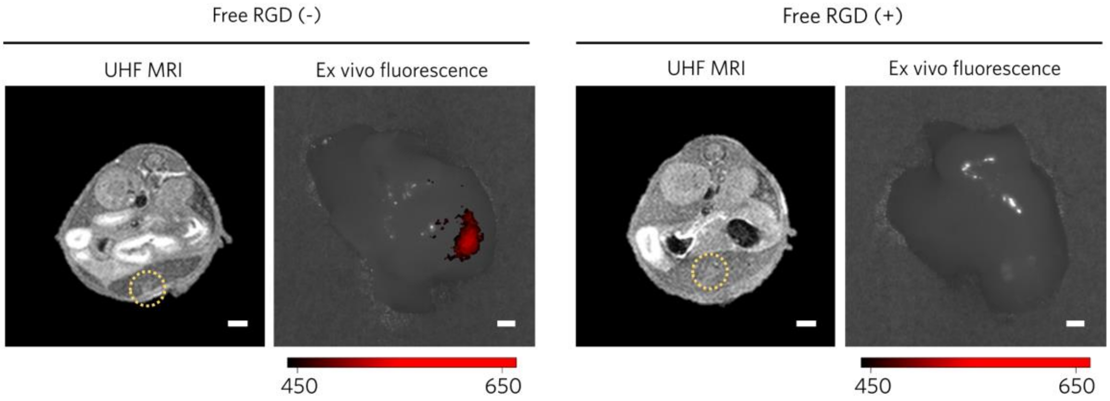

Supplementary Figure 14. Competitive study of PMNP-RGD and free RGD peptide. The UHF MRI of primary hepatic tumour and ex vivo fluorescence imaging of the liver tissue at 5 h post single injection of RITC labelled PMNP-RGD or co-injection of RITC labelled PMNP-RGD and free RGD peptide (Scale bar = 2 mm).

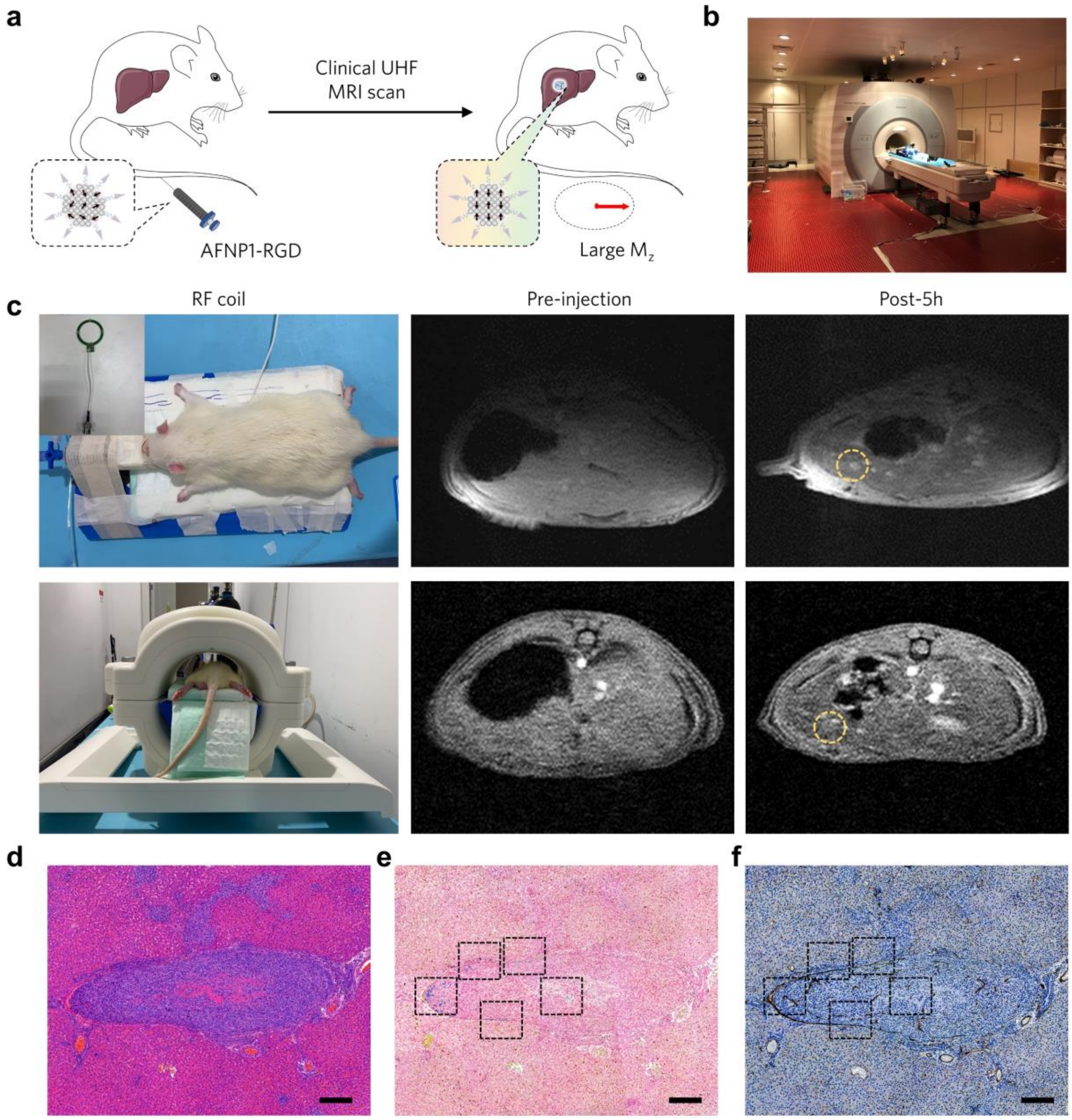

Supplementary Figure 15. UHF MRI of microscopic primary tumour using AFNP1 in rat. a, Schematic illustration of AFNP1-RGD enhanced MRI of hepatic microscopic primary tumours using a clinical UHF MRI scanner. **b,** Photograph of the clinical 7 T MRI scanner. **c,** T1-weighted images of the primary hepatic tumour in the rat before and 5 h after i.v. injection of AFNP1-RGD. Upper panel, animal surface radiofrequency (RF) receiver coil; lower panel, clinical knee RF receiver coil. Yellow circles indicate the tumour. **d,** H&E, **e,** Prussian blue, and **f**. CD31 immunochemistry staining of the tumour shown in **c,** CD31 positive angiogenic sites are stained as brown in **f** (Scale bar = 200 μm). In **d-f** experiments were repeated three times independently.

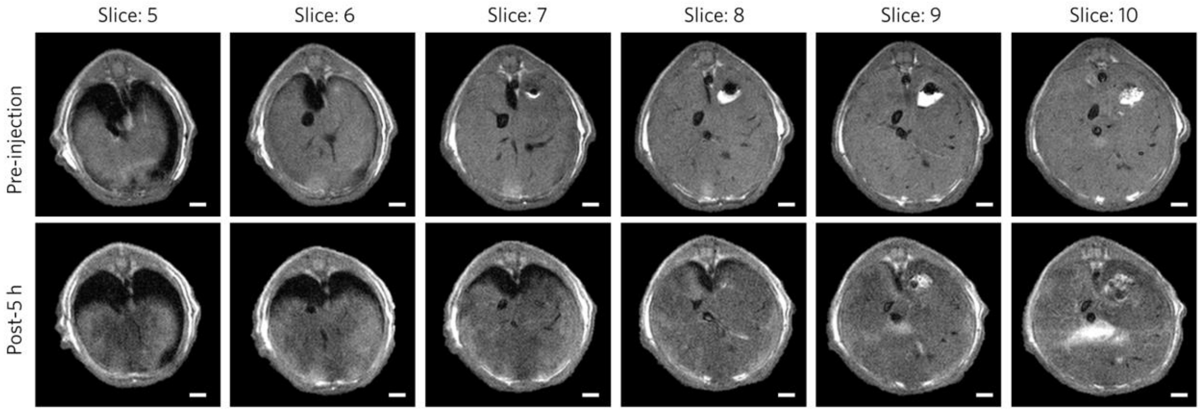

Supplementary Figure 16. UHF MRI of micrometastases using PMNP-RGD. T1-weighted MR images of mice bearing hepatic micrometastases before and at 5 h post injection of PMNP-RGD (scale bar = 2 mm).

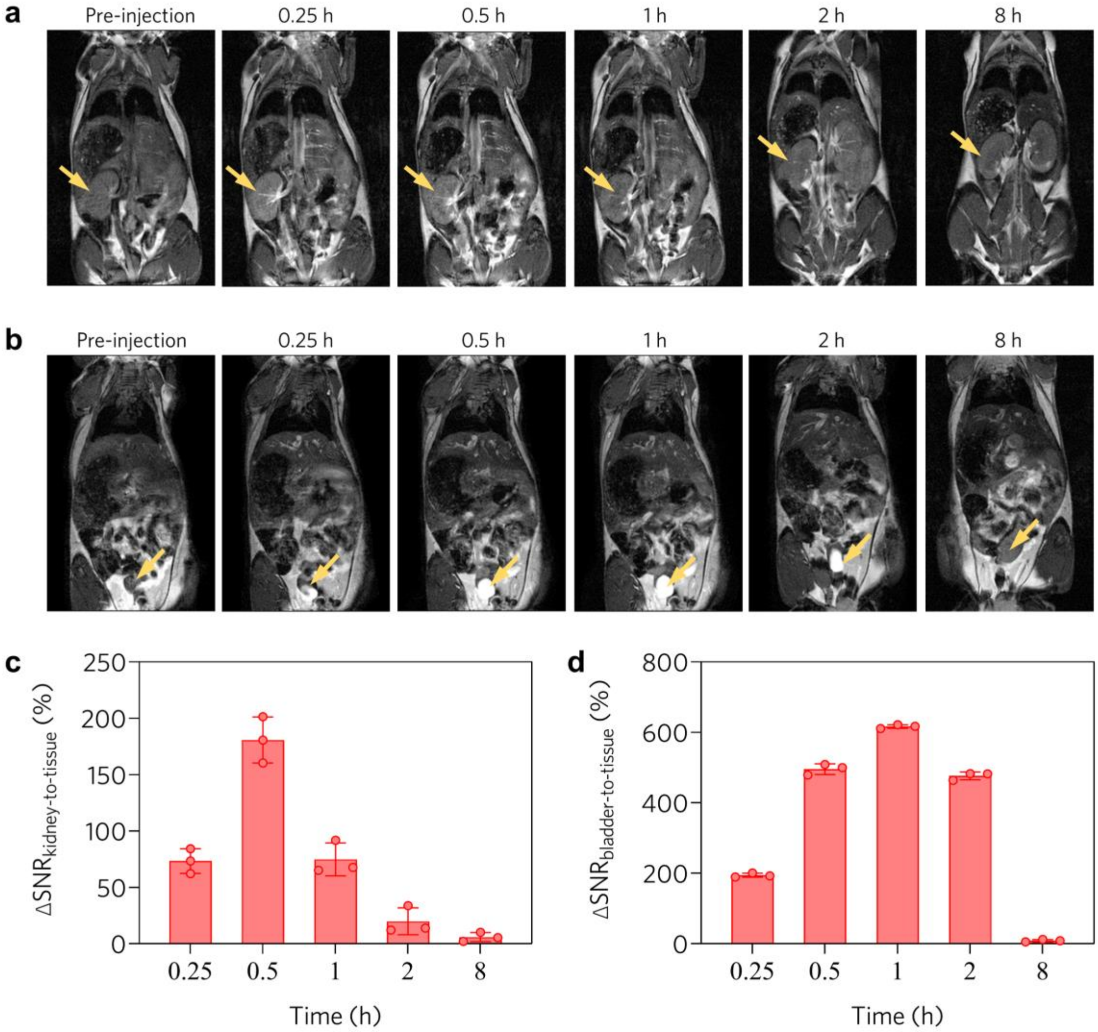

Supplementary Figure 17. In vivo clearance analysis of AFNP1-RGD. a,b, T1-weighted MR images before and at 0.25, 0.5, 1, 2, 8 h post injection of AFNP1-RGD (5 mg Fe kg^{-1}). **c,d,** Quantification of SNR changes of (**c**) kidney, and (**d**) bladder. n = 3 independent animals. Data are presented as mean ± SD. Source data are provided as a Source Data file.

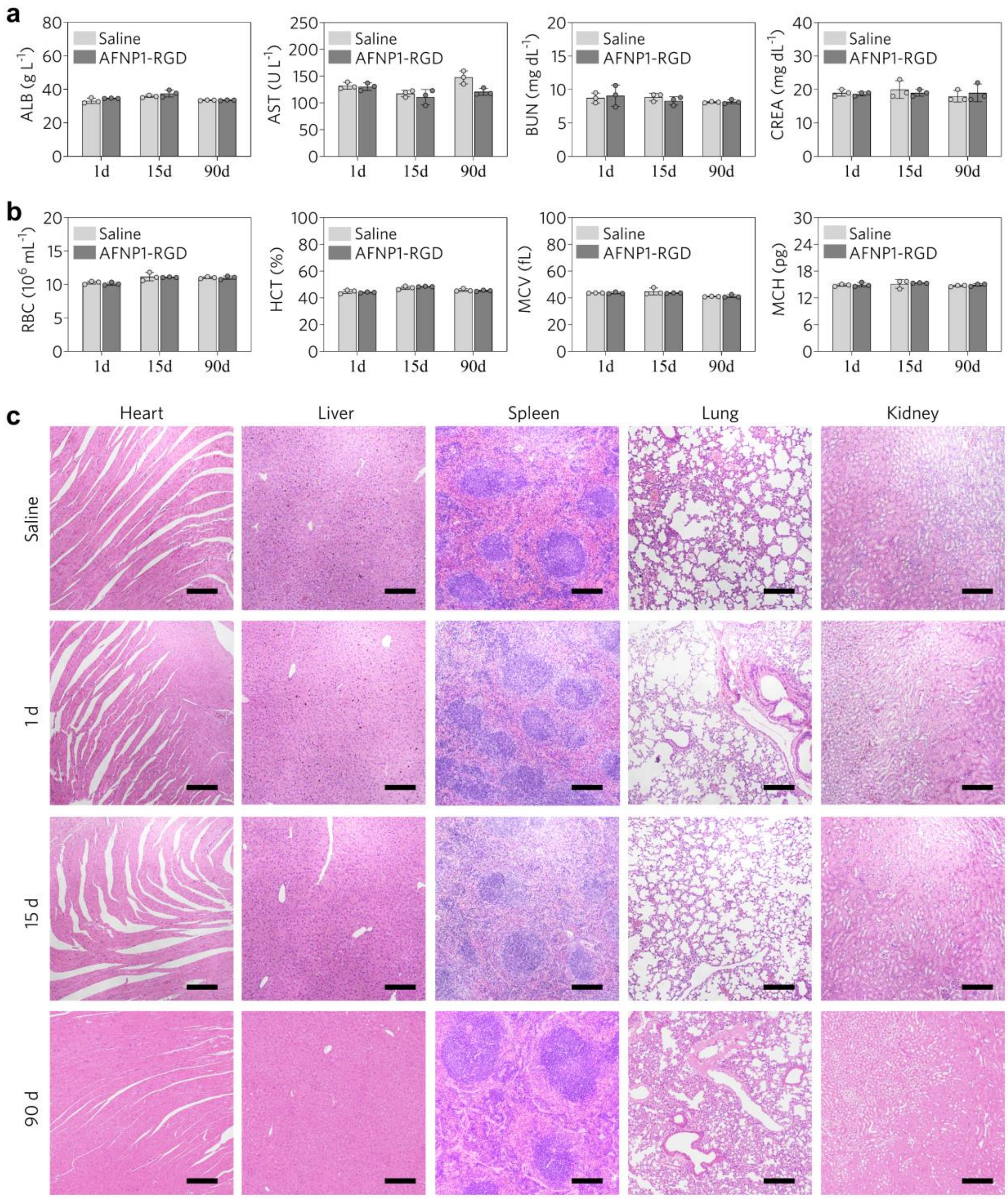

Supplementary Figure 18. In vivo toxicological analysis of AFNP1-RGD. Balb/c female mice (6-8 weeks) were intravenously injected with 200 µl of saline or AFNP1-RGD at a dose of 5.0 mg Fe/kg. **a,** Blood biochemistry data for liver function markers (ALB, AST) and kidney function markers (BUN, CREA). *n* = 3 independent animals. Data are presented as mean ± SD. **b,** Hematological tests including red blood cell (RBC), hematocrit (HCT), mean corpuscular volume (MCV), and mean corpuscular hemoglobin (MCH). *n* = 3 independent animals. Data are presented as mean ± SD. **c,** Morphology of main viscera (heart, liver, spleen, lung, and kidney) after treatment with saline or AFNP1-RGD in 1-, 15- and 90-days; Scale bar = 200 µm. Source data are provided as a Source Data file. In **c** experiments were repeated three times independently.

Supplementary Tables:

Supplementary Table 1. Summary of Fe/Pt molar ratios of AFNPs.

Nanoprobe	Fe/Pt molar ratio
AFNP1	0.3121
AFNP2	0.3335
AFNP3	0.3357
AFNP4	0.3317
AFNP5	0.3427

Supplementary Table 2. Summary of primary hepatic tumour detection with contrast agents enhanced MRI

Contrast agent	Tumour size (mm)	Magnetic field (T)	Tumour model	Ref.
Den-Apt1 (Gd^{3+} binding with G5-dendrimer)	3-4	7	Primary hepatic tumour	1
RGD modified Fe_3O_4	2.2	3	Primary hepatic tumour	2
AFNP1-RGD	0.6	9	Primary hepatic tumour	This work

Supplementary Table 3. Summary of metastatic tumour detection by contrast agents enhanced MRI

Contrast agent	Tumour size (mm)[a]	Magnetic field (T)	Tumour model	Ref.
Mn-PyC3A-derived complexes	1.30	4.7	Hepatic metastasis in colorectal cancer	3
CREKA-Tris (Gd)DOTA)$_3$	0.50	7	Bone metastasis in breast cancer	4
Iron oxide nanochain	0.50	9.4	Hepatic metastasis in breast cancer	5
Zn^{2+} doped iron oxide nanoparticle	0.40	7	Metastatic liver tumour	6
UMFNP-CREKA (manganese ferrite nanoparticle)	0.39	7	Lung metastasis in breast cancer	7
ProCA32.CXCR4	0.27[b]	7	Hepatic metastases	8
AFNP1-RGD	0.20	9	Hepatic metastases	This work

[a]Tumour size is the minimum detection size in the reference. [b]The smallest tumour detected in this study is 0.01 mm^3 in volume, which is converted to ~ 0.27 mm in size assuming the tumour has a spherical shape.

Supplementary Table 4. Summary of correlation between tumour sizes detected by contrast enhanced-MRI and H&E histology

Contrast agent	correlation[a]	Magnetic field (T)	Tumour model	Ref.
ProCA32	0.920	4.7	Hepatic metastases	9
ProCA32.CXCR4	0.968	7	Hepatic metastases	8
AFNP1-RGD	0.989	9	Hepatic metastases	This work

Supplementary Table 5. Summary of tumour detection sensitivity of current clinical imaging modalities when using histopathology as the reference standard.

Contrast agent	Tumour size (mm)	Sensitivity (%)	Imaging modalities	Tumour type	Ref.
Superparamagnetic nanoparticles	< 5	41.1	MRI (1.5 T)	Lymph-node metastases in Prostate Cancer	10
Gd-DTPA	< 5	41.2	MRI (1.5 T)	Hepatic metastases	11
Gd-DOTA	< 5	42.9	MRI (1.5 T)	Hepatic metastases	12
Gastrografin	< 5	43	CT[a]	Peritoneal carcinomatosis	13
AFNP1-RGD	< 5	90.2	MRI (9 T)	Hepatic metastases	This work

[a]CT: computed tomography.

References

1. Yan, H. *et al*. Imaging tiny hepatic tumor xenografts via endoglin targeted paramagnetic/optical nanoprobe. *ACS Appl. Mater. Inter.* **10**, 17047-17057 (2018).
2. Jia, Z. *et al*. Active-target T1-weighted MR imaging of tiny hepatic tumor via RGD modified ultra-small Fe_3O_4 nanoprobes. *Theranostics* **6**, 1780-1791 (2016).
3. Wang, J. *et al*. Manganese-based contrast agents for magnetic resonance imaging of liver tumors: structure activity relationships and lead candidate evaluation. *J. Med. Chem.* **61**, 8811-8824 (2018).
4. Zhou, Z. *et al*. MRI detection of breast cancer micrometastases with a fibronectin-targeting contrast agent. *Nat. Commun.* **6**, 1-11 (2015).
5. Peiris, P. M. *et al*. Imaging metastasis using an integrin-targeting chain-shaped nanoparticle. *ACS Nano* **6**, 8783-8795 (2012).
6. Zhao, Z. *et al*. Cation exchange of anisotropic-shaped magnetite nanoparticles generates high-relaxivity contrast agents for liver tumor imaging. *Chem. Mater.* **28**, 3497-3506 (2016).
7. Ref. 31 in the main text.
8. Ref. 48 in the main text.
9. Xue, S. *et al*. Protein MRI contrast agent with unprecedented metal selectivity and sensitivity for liver cancer imaging. *Proc. Natl. Acad. Sci. U. S. A* **112**, 6607-6612 (2015).
10. Harisinghani, M. G. *et al*. Noninvasive detection of clinically occult lymph-node metastases in prostate cancer. *N. Engl. J. Med.* **348**, 2491-2499 (2003).

11. Hardie, A. D. *et al*. Diagnosis of liver metastases: value of diffusion-weighted MRI compared with gadolinium-enhanced MRI. *Eur. Radiol.* **20**, 1431-1441 (2010).
12. Soyer, P. *et al*. Preoperative detection of hepatic metastases: Comparison of diffusion-weighted, T2-weighted fast spin echo and gadolinium-enhanced MR imaging using surgical and histopathologic findings as standard of reference. *Eur. J. Radiol.* **80**, 245-252 (2011).
13. Marin, D. *et al*. 64-Section multi-detector row CT in the preoperative diagnosis of peritoneal carcinomatosis: correlation with histopathological findings. *Abdom. Imaging* **35**, 694-700 (2010).
