# Supplementary material for: Artificially engineered antiferromagnetic nanoprobes for ultra-sensitive histopathological level magnetic resonance imaging
Source: Nat Commun. 2021 Jun 22;12:3840. doi: 10.1038/s41467-021-24055-2 (PMC8219830; doi:10.1038/s41467-021-24055-2)
Supplement: Supplementary file 3 — Reporting Summary [file 41467_2021_24055_MOESM3_ESM.pdf]

nature research

Corresponding author(s): Daishun Ling

Last updated by author(s): May 14, 2021

Reporting Summary

Nature Research wishes to improve the reproducibility of the work that we publish. This form provides structure for consistency and transparency in reporting. For further information on Nature Research policies, see our Editorial Policies and the Editorial Policy Checklist.

Statistics

For all statistical analyses, confirm that the following items are present in the figure legend, table legend, main text, or Methods section.

n/a	Confirmed	
☐	☒	The exact sample size (n) for each experimental group/condition, given as a discrete number and unit of measurement
☐	☒	A statement on whether measurements were taken from distinct samples or whether the same sample was measured repeatedly
☐	☒	The statistical test(s) used AND whether they are one- or two-sided<br>*Only common tests should be described solely by name; describe more complex techniques in the Methods section.*
☒	☐	A description of all covariates tested
☒	☐	A description of any assumptions or corrections, such as tests of normality and adjustment for multiple comparisons
☐	☒	A full description of the statistical parameters including central tendency (e.g. means) or other basic estimates (e.g. regression coefficient) AND variation (e.g. standard deviation) or associated estimates of uncertainty (e.g. confidence intervals)
☐	☒	For null hypothesis testing, the test statistic (e.g. F, t, r) with confidence intervals, effect sizes, degrees of freedom and P value noted<br>*Give P values as exact values whenever suitable.*
☒	☐	For Bayesian analysis, information on the choice of priors and Markov chain Monte Carlo settings
☒	☐	For hierarchical and complex designs, identification of the appropriate level for tests and full reporting of outcomes
☒	☐	Estimates of effect sizes (e.g. Cohen's d, Pearson's r), indicating how they were calculated

Our web collection on statistics for biologists contains articles on many of the points above.

Software and code

Policy information about availability of computer code

Data collection	The TEM images were acquired on a transmission electron microscope (Hitachi HT7700, Japan). HRTEM images were obtained on a transmission electron microscope (FEI Tecnai G2 F20 S-TWIN, U.S.A). Dynamic light scattering (DLS) measurements were conducted on a Nano ZS instrument (Malvern, U.K.). XRD patterns were obtained by using an X-ray diffractometer (PANalytical B.V. X-pert Powder, Netherlands). Magnetic measurements were performed by a superconducting quantum interference device (SQUID) magnetometer (Cryogenic J3426, U.K.). Fourier-transform infrared spectra (FT-IR) were obtained by an infrared spectrophotometer (JASCO FI/IR-4100, Japan). XPS analysis was acquired via an XPS system (Thermo Scientific ESCALAB 250 Xi, U.K.). MRI images were obtained by a 9 T MRI scanner (Timemedical 9 T/110, U.S.A). The amounts of Fe in samples were measured by inductively coupled plasma mass spectrometry (ICP-MS, PerkinElmer NexION 300X, U.S.A). The fluorescence images were acquired by the VISQUE InVivo Elite imaging system (Vieworks, Korea).
Data analysis	Data analysis was performed by using OriginPro (version 8.5.0 SR1), Microsoft Excel (version 16.0.13929.20206), GraphPad Prism (version 8.0.2), Image J (version 1.52a), MATLAB (version R2017b), 3DSlicer (version 4.11.0), RadiAnt DICOM Viewer (version 2020.2.3), CaseViewer (version 2.0), Gatan DigitalMicrograph (version 2.10.1282.0).

For manuscripts utilizing custom algorithms or software that are central to the research but not yet described in published literature, software must be made available to editors and reviewers. We strongly encourage code deposition in a community repository (e.g. GitHub). See the Nature Research guidelines for submitting code & software for further information.

Data

Policy information about availability of data

All manuscripts must include a data availability statement. This statement should provide the following information, where applicable:

- Accession codes, unique identifiers, or web links for publicly available datasets
- A list of figures that have associated raw data
- A description of any restrictions on data availability

The data is available within the article, supplementary information or available from the authors upon request. The source data underlying Fig. 2b, 2e, 2f, 3c-e, 3i, 3j, 4f, 5e-g, as well as Supplementary Figs. 1-5, 6b-f, h-j, 7b-d, 8, 9, 12, 13b-h, 17c, 17d, 18a, 18b are provided as a Source Data file. Source data are provided with this paper.

Field-specific reporting

Please select the one below that is the best fit for your research. If you are not sure, read the appropriate sections before making your selection.

[x] Life sciences [ ] Behavioural & social sciences [ ] Ecological, evolutionary & environmental sciences

For a reference copy of the document with all sections, see nature.com/documents/nr-reporting-summary-flat.pdf

Life sciences study design

All studies must disclose on these points even when the disclosure is negative.

Sample size	Sample size choice was based on previous studies (ref. Shin, T. et al. 2021, https://doi.org/10.1038/s41551-021-00687-z; Yu, B. et al. 2020, https://doi.org/10.1038/s41467-020-17380-5; Li, Y. et al. 2017, https://doi.org/10.1038/ncomms15653), not predetermined by a statistical method. Sample sizes were indicated in the legend of each Figure and Supplementary Figure.
Data exclusions	No data were excluded.
Replication	We confirm all attempts at replication were successful. All the experimental findings were all replicated at least 3 times.
Randomization	All samples were randomly allocated into experimental groups.
Blinding	The operators responsible for statistical data analysis were blinded and unaware of group allocation throughout the experiments. In the experiments of in vivo UHF MRI, ex vivo fluorescence imaging, H&E staining, Prussian blue staining and CD31 immunohistochemical staining, the experienced radiologists or pathologists were blinded to determine the tumour regions.

Reporting for specific materials, systems and methods

We require information from authors about some types of materials, experimental systems and methods used in many studies. Here, indicate whether each material, system or method listed is relevant to your study. If you are not sure if a list item applies to your research, read the appropriate section before selecting a response.

Materials & experimental systems

n/a	Involved in the study	
[x]	[ ]	Antibodies
[ ]	[x]	Eukaryotic cell lines
[x]	[ ]	Palaeontology and archaeology
[ ]	[x]	Animals and other organisms
[x]	[ ]	Human research participants
[x]	[ ]	Clinical data
[x]	[ ]	Dual use research of concern

Methods

n/a	Involved in the study	
[x]	[ ]	ChIP-seq
[x]	[ ]	Flow cytometry
[x]	[ ]	MRI-based neuroimaging

Eukaryotic cell lines

Policy information about cell lines

Cell line source(s)	Walker 256 cells and RAW264.7 cells were purchased from American Type Culture Collection (ATCC, Manassas, VA). Huh7-GFP-Luc cells were purchased from OBiO Technology (Shanghai) Corp., Ltd. (Shanghai, China).
Authentication	No further authentication was done after the cells were obtained from the vendors.
Mycoplasma contamination	The cell line was tested negative for mycoplasm contamination per suppliers.
Commonly misidentified lines (See ICLAC register)	No commonly misidentified lines were used.

Animals and other organisms

Policy information about studies involving animals; ARRIVE guidelines recommended for reporting animal research

Laboratory animals	BALB/c nude mice (Male, 4-5 weeks), BALB/c mice (Male, 4-6 weeks) and Wistar rat (Male, 150-200 g) were purchased from Shanghai SLAC Laboratory Animal Co., Ltd. and were housed in open top caging in a 14-10 h light-dark cycle and maintained a room temperature of 25°C with 40-60% humidity.
Wild animals	Wild animals were not involved in this study.
Field-collected samples	Field-collected samples were not involved in this study.
Ethics oversight	The use and care of the mice were in accordance with the guidelines of the Institutional Animal Care and Use Committee (IACUC) of Zhejiang University. All procedures were approved by the IACUC of Zhejiang University.

Note that full information on the approval of the study protocol must also be provided in the manuscript.
