# Supplementary material for: Artificially engineered antiferromagnetic nanoprobes for ultra-sensitive histopathological level magnetic resonance imaging
Source: Nat Commun. 2021 Jun 22;12:3840. doi: 10.1038/s41467-021-24055-2 (PMC8219830; doi:10.1038/s41467-021-24055-2)
Supplement: Supplementary file 2 — Peer Review File [file 41467_2021_24055_MOESM2_ESM.pdf]

Editorial Note: This manuscript has been previously reviewed at another journal that is not operating a transparent peer review scheme. This document only contains reviewer comments and rebuttal letters for versions considered at Nature Communications.

REVIEWERS' COMMENTS

Reviewer #2 (Remarks to the Author):

The authors have made further constructive changes to their manuscript which I have seen in previous form. I had commented on the last submission noting some relatively minor issues around clarity of the work. Specifically the differences in data collection regimes (signal averaged) between scans on their mouse scanner versus their data collected on a human scanner and also issues around their comparison of the sensitivity of their probe against other data in the literature.

The authors have now addressed both of these comments and have added additional information and explanation in the revised text, including data in a supplementary table regarding performance against other published agents.

Andrew Blamire

Reviewer #3 (Remarks to the Author):

I don't have any further comments. It should be fine for publishing.
